# Curing Behavior of UV-Initiated Surface-Modified Nano-TiO_2_/Epoxy Resin Prepolymers and the Properties of Cured Composites

**DOI:** 10.3390/polym15071756

**Published:** 2023-03-31

**Authors:** Renkun Xia, Jiaojiao Xi, Zhiyun Zhang, Yannan He, Zhiqiang Yu

**Affiliations:** 1 Department of Chemistry, Fudan University, Shanghai 200433, China; 2 Department of Material Science, Fudan University, Shanghai 200433, China

**Keywords:** nano-TiO_2_, surface modification, coupling agents, epoxy resin matrix composites, UV-induced curing, isothermal curing kinetics

## Abstract

Nano-titanium dioxides (nano-TiO_2_) surface modified with isopropyl tri(dioctylpyrophosphate) titanate (NDZ-201), a titanate coupling agent, and 3-glycidoxypropyltrimethoxysilane (KH-560), a silane coupling agent, were separately mixed with bisphenol A epoxy resin (DEGBA) prepolymer and then cured using a UV-normal temperature synergistic curing process. Then, the isothermal curing process of the system was investigated by differential scanning calorimetry (DSC). The relationship between the organization structures, mechanical properties, and heat resistance properties of the cured composites and material formulation was studied, and the DSC results showed that the addition of nano-TiO_2_ reduced the curing reaction rate constant *k_1_* and increased the *k_2_* of the prepolymer, while the activation energy of the curing reaction after UV irradiation *E*_a1_ decreased, and the activation energy in the middle and later periods *E*_a2_ increased. The characterization results of the composite material showed that nano-TiO_2_ as a scattering agent reduced the photoinitiation efficiency of UV light, and due to its obvious agglomeration tendency in the epoxy resin, the mechanical properties of the composite material were poor. The dispersibility of the coupling-agent-modified nano-TiO_2_ in the epoxy resin was greatly enhanced, and the mechanical and heat resistance properties of the composite material improved remarkably. The comparison results of the two coupling agents showed that NDZ-201 had better performance in increasing the impact strength by 6.8% (minimum value, the same below) and the maximum thermal decomposition rate temperature by 4.88 °C of the composite, while KH-560 improved the tensile strength by 7.3% and the glass transition temperature (*T_g_*) by 3.34 °C of the composite.

## 1. Introduction

Epoxy resins are widely used in paints and coatings, adhesives, electronic materials, and the aerospace industry due to their ease of processing, high safety, excellent solvent and chemical resistance, and excellent adhesion to many substrates [1,2,3]. However, epoxy resins have relatively minimal resistance to crack initiation and expansion due to a highly cross-linked network structure, which limits their application in many scenarios [4]. To improve the physical properties of epoxy resins, a variety of epoxy-resin-based composites have been prepared by combining various inorganic/organic materials as tougheners or reinforcing agents with epoxy resins [5,6,7].

Nano-TiO_2_ is commonly used to toughen the epoxy matrix due to its high stability and good optical properties [8,9]. Amit et al. [10] investigated the relationship between the loading percentage of nano-TiO_2_ and the elastic modulus, flexural modulus, energy storage modulus, *T_g_*, and thermal stability of the composites. Goyat et al. [11] revealed the toughening mechanism of nano-TiO_2_ on the epoxy matrix by studying the field emission scanning electron microscope (FESEM) images of nano-TiO_2_/epoxy-resin-based composites. However, due to their high surface energy, nano-TiO_2_ particles tend to agglomerate in the epoxy matrix, decreasing the cross-linking density of the system, which will adversely affect the properties of the composite. Surface modification techniques have often been used to reduce the surface energy of nano-TiO_2_ particles to improve the interactions between the nanoparticle and the polymer matrix. As a result, durable chemical connections between the two incompatible phases are built [12,13,14,15,16,17,18]. Coupling agents have commonly been used for the modification of various surfaces, such as the surfaces of nano-TiO_2_ particles. Mallakpour et al. [19] reported on the surface modification of nano-TiO_2_ with 3-aminopropyltriethoxysilane (APTES) and found that the thermal stability of the composite improved due to a better dispersion of the modified TiO_2_ in the epoxy matrix. Tangchantra et al. [20] investigated the modification effects of different silane coupling agents grafted on nano-TiO_2_ surfaces, including cetyltrimethoxysilane (HTMS), triethoxyvinylsilane (TEVS), and aminopropyltrimethoxysilane (APS). The results showed that the silane coupling agents could modify the particle surfaces by hydrolytic condensation of titanium isopropoxide. Chen et al. [21] reported the cross linking and chemical bonding mechanism of 3-aminopropyltrimethoxysilane (APTMS) and 3-isocyanatopropyltrimethoxysilane (IPTS) on nano-TiO_2_ particle surfaces. Zhang et al. [22] pretreated the nano-TiO_2_ surface with 3-aminopropyltriethoxysilane (KH-550) to prepare poly(lactic-co-glycolic acid) (PLGA)/TiO_2_ organic–inorganic hybrid materials, which have been widely used as a nanodrug sustained-release carrier. As a result, KH-550 expanded the use of nano-TiO_2_ in this case.

Previous studies related to the surface modification of nano-TiO_2_ by coupling agents have mainly focused on the influence of silane coupling agents on nano-TiO_2_/epoxy resin composites, with few reports on the surface modification of nano-TiO_2_ by other types of coupling agents, such as titanate coupling agents. In addition, there is a lack of studies on the effect of coupling agents during the curing process of nano-TiO_2_/epoxy resin prepolymers, and the conditions of the curing process under UV irradiation have rarely been reported.

In this study, nano-TiO_2_ particles modified with NDZ-201 and KH-560 were prepared using the solvothermal method, and the effect and mechanism of modification were characterized and discussed. Then, the nano-TiO_2_/epoxy resin prepolymers were cured by a UV-normal temperature synergistic curing process to obtain various types of nano-TiO_2_/epoxy resin composites. The isothermal curing kinetics of the system were studied, and the microstructure, mechanical, and heat resistance properties of the composites were characterized and discussed.

## 2. Materials and Methods

### 2.1. Preparation of Surface-Modified Nano-TiO_2_

The nano-TiO_2_ was added to a toluene solvent and dispersed ultrasonically for 1 h. Then, different proportions of NDZ-201 and KH-560 (10, 20, 30, 40, and 50 wt% relative to nano-TiO_2_) were added to the suspension, and a modification reaction was carried out at a stirring rate of 750 rpm at 60 °C for 2 h. The solvent was removed by rotary evaporation at a temperature of 70 °C and a vacuum degree of 0.1 MPa. After washing and precipitating several times, the precipitate was placed into a vacuum oven and dried for 24 h at a temperature of 100 °C and a vacuum degree of 0.1 MPa to obtain a series of modified nano-TiO_2_ particles.

**Figure 1:**
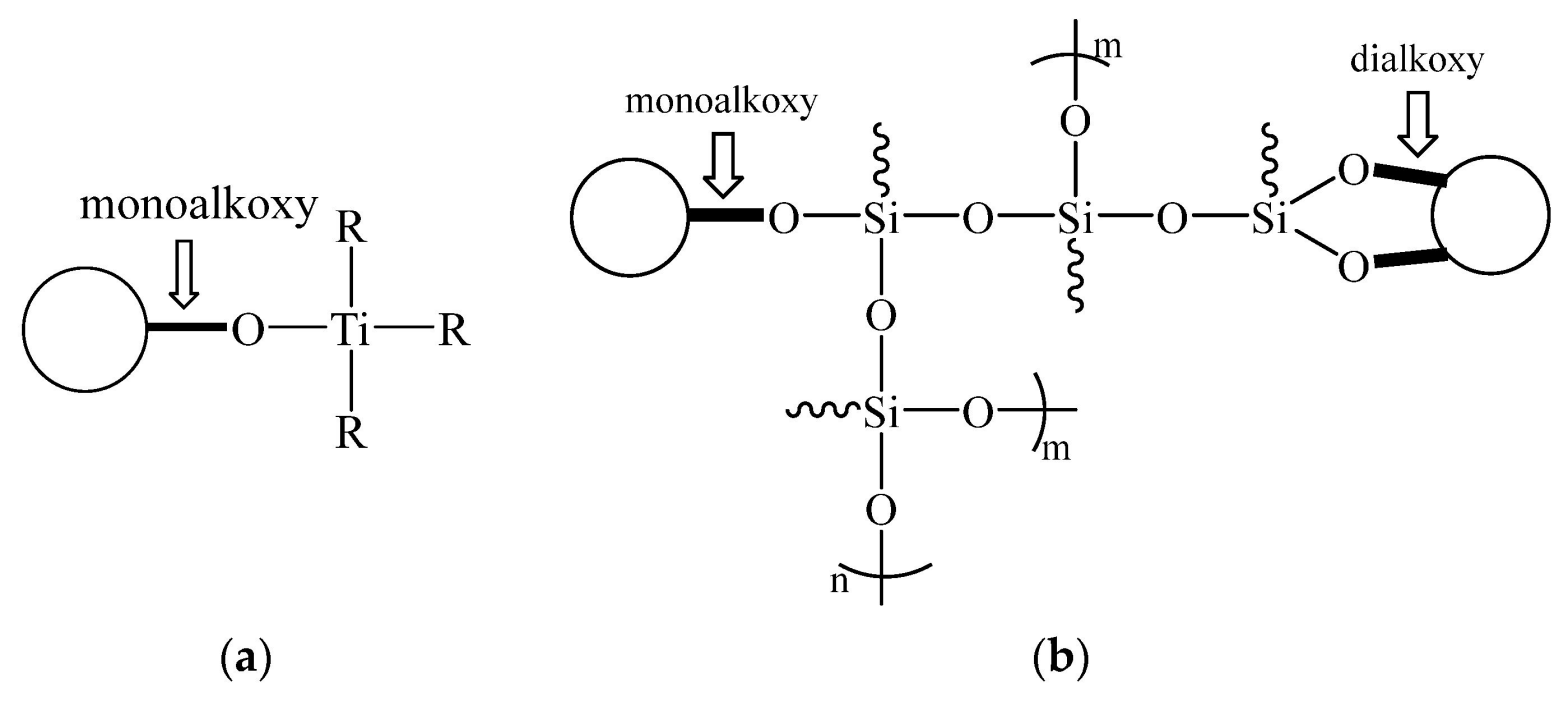
Nano-TiO_2_ particle structure after surface modification of (**a**) NDZ-201 and (**b**) KH-560 [23].

The structure of the modified nano-TiO_2_ particles is shown in Figure 1 [23]. Each NDZ-201 molecule was connected to the surface of TiO_2_ with a single bond, and each KH-560 molecule was connected to the TiO_2_ surface by 1–2 bonds, with a possible O–Si–O bridge between two nano-TiO_2_ particles.

### 2.2. Preparation of Nano-TiO_2_/Epoxy Resin Prepolymers and Composites

Nano-TiO_2_ and modified nano-TiO_2_ (modified by 30 wt% NDZ-201 and KH-560, respectively, relative to nano-TiO_2_) were mixed into E-51 with an epoxy equivalent of 0.48~0.54 at a proportion of 1 wt% (relative to E-51), respectively, and stirred at 140 °C and 1000 rpm for 2 h. Four prepolymer systems were obtained, which included the pure epoxy resin prepolymer (abbreviated as EP), the nano-TiO_2_/epoxy resin prepolymer (abbreviated as TEP), the NDZ-201-modified nano-TiO_2_/epoxy resin prepolymer (abbreviated as NTEP), and the KH-560-modified nano-TiO_2_/epoxy resin prepolymer (abbreviated as KTEP).

**Figure 2:**
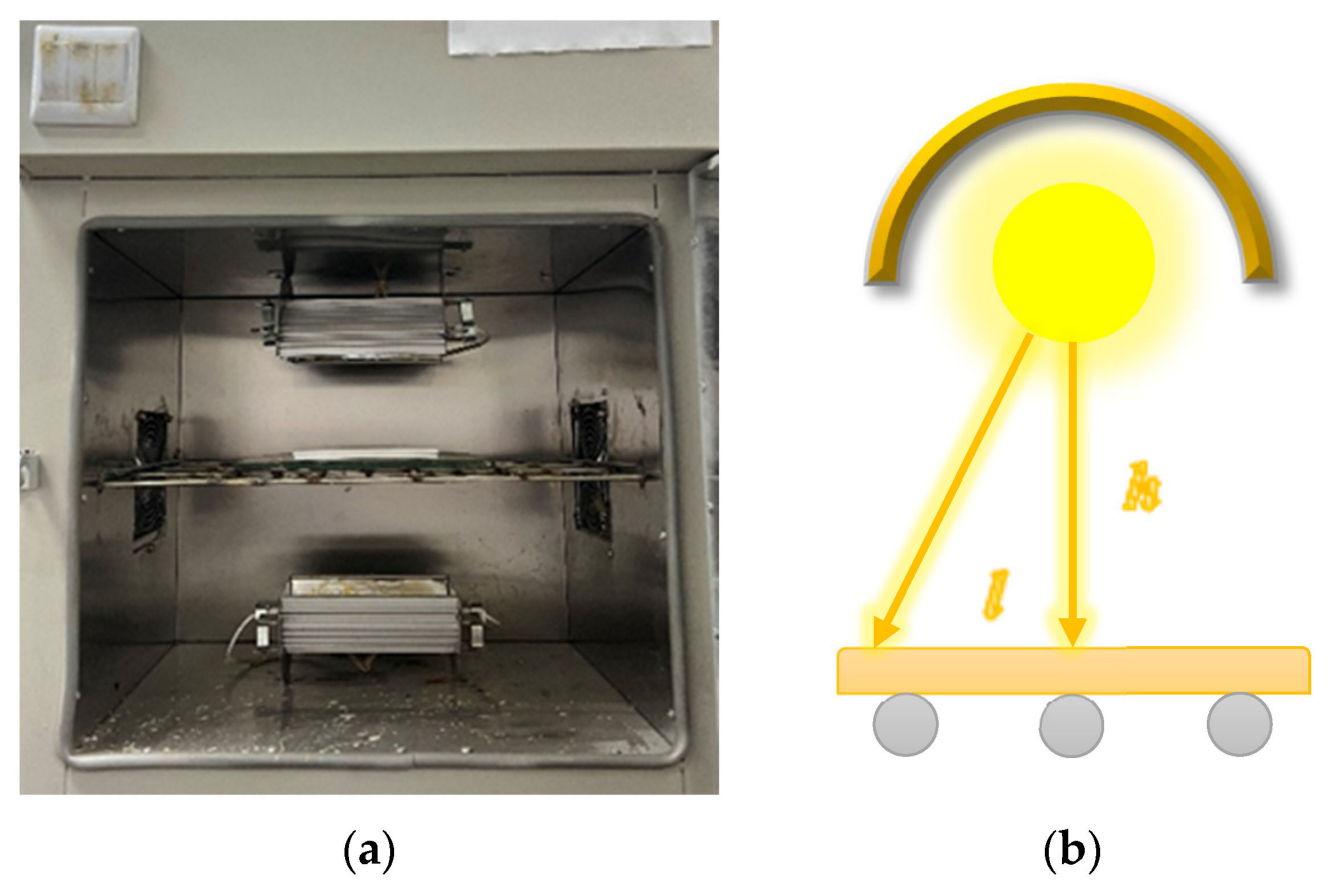
(**a**) Photo of UV light box and (**b**) cross-section schematic diagram of light box.

The 4 prepolymer systems were cooled to about 60 °C, and then 4 wt% (relative to E-51) of triarylsulfonium hexafluoroantimonate salts and 15 wt% (relative to E-51) of triethylenetetramine (TETA) were added. The mixtures were poured into a curing mold (the depth of the mold was 4 mm); placed in a 1000 W UV light box to cure under UV irradiation times of 30 s, 45 s, 60 s, and 75 s; and then cooled for 60 s. This UV irradiation–cooling cycle was repeated three times. The photo of the UV light box is shown in Figure 2a. When the light box was working, the upper light was turned on, the lower light was off, and the power of the single lamp was *P* = 1000 W. The schematic diagram of the light box’s section is shown in Figure 2b.

According to Figure 2b, the upper part of the lamp source was covered by a semi-cylindrical reflective shell, and the lower part was facing the mold placed on the iron frame. The vertical distance of the lamp source from the mold was measured as *h* = 15 cm. The maximum distance from the center of the mold to the sample slots on both sides was *l* = 7 cm. It was considered that the irradiation intensity at the position equidistant from the lamp source was equal. The irradiation intensity *E_r_* of the point with a distance *r* from the lamp source is shown in Equation (1).

(1) $$E_{r}=\frac{P}{{2\pi r}^{2}}$$

Under the experimental conditions, *E_r_* of the center of the mold was at the maximum, while that of both sides was at the minimum. The range of the irradiation intensity *E* is shown in Equation (2).

(2) $$\frac{P}{{2\pi (h}^{2}{+l}^{2})}<E<\frac{P}{{2\pi h}^{2}}$$

According to Equation (2), the range of *E* was 5.8 kW/m^2^~7.1 kW/m^2^, which meant that the difference in irradiation intensity among different areas of the mold under the same irradiation conditions was within 22%. Under the conditions of an irradiation time of 30 s, 45 s, 60 s, and 75 s, respectively, the irradiation energy range of the mold is shown in Table 1. According to Table 1, there was no overlap in the irradiation energy range of the mold under different irradiation times. The experimental results had a certain degree of differentiation.

After UV irradiation, curing was continued at room temperature for 2 h to obtain the composite splines of each system, and the curing mold sizes were implemented in accordance with the GB/T 1040.1–2018 and GB/T 1843–2008 standards. The relationship between the sample code and the curing parameters is summarized in Table 2.

### 2.3. Characterization and Testing

The absorption spectrum of the modified nano-TiO_2_ was tested by a Nicolet 6700 in situ Fourier transform infrared (FT-IR) spectrometer (Thermo Fisher, Calsbad, CA, USA) in the range of 4000~400 cm^−1^. The average particle size of nano-TiO_2_ in anhydrous ethanol at 25 °C dispersed at a ratio of 1 wt% (relative to absolute ethanol, respectively) was tested by a Zetasizer Nano ZS laser particle sizer (Malvern Panalytical Ltd., Malvern, UK). The surface elemental absorption of the modified TiO_2_ nanoparticles was analyzed by a 5000C X-ray photoelectron spectrometer (Physical Electronics, Chanhassen, MN, USA).

The viscosities of the EP, TEP, NTEP, and KTEP prepolymers were tested by an SNB-2 digital rotational viscometer (Kejing, Shenzhen, China) at 25 °C, where the humidity of the test environment was 40%, and the instrument speed (shear rate) was 12.0 rpm. The isothermal curing heat release rates of the four prepolymer systems during the UV-normal temperature synergistic curing process were tested by a Q2000 differential scanning calorimeter (TA Instruments, New Castle, DE, USA).

The surface morphologies of the composites were observed by a VEGA 3 XMU scanning electron microscope (TESCAN Brno s.r.o., Brno-Kohoutovice, Brno, Czech Republic), with an accelerating voltage of 5.0 kV. A thin layer of gold was sprayed on the sample surfaces to reduce charge accumulation.

The composite impact strength was tested by a JJ-TEST instrument impact tester (ZwickRoell GmbH & Co. KG, Ulm, Germany), according to requirements of the GB/T 1843–2008 standard, where the gap width of the samples was 2 mm, and the impact energy was 5~500 J. The tensile strength and elastic modulus of the composites were tested by an NANC universal testing machine (Wance, Shenzhen, China), according to the requirements of the GB/T 1040.1–2018 standard. The loading rate during the test was 2 mm/min.

*T_g_* of the material was tested by a Q2000 differential scanning calorimeter (TA Instruments, New Castle, DE, USA) with a temperature range of 20~200 °C in nitrogen. In the test, the material was first heated at a heating rate of 5 °C/min to eliminate internal stress, cooled to 20 °C, and then heated again at the same heating rate. The thermal decomposition process of the materials was analyzed by a TGA8000 thermal gravimetric analyzer (PerkinElmer, Shelton, CT, USA) at a temperature range of 20~800 °C in air. The heating rate during the test was 10 °C/min. The mass of the samples was around 5 g. Each sample was a single block before the test.

## 3. Results and Discussion

### 3.1. Characterization of the Modified TiO_2_ Nanoparticles

**Figure 3:**
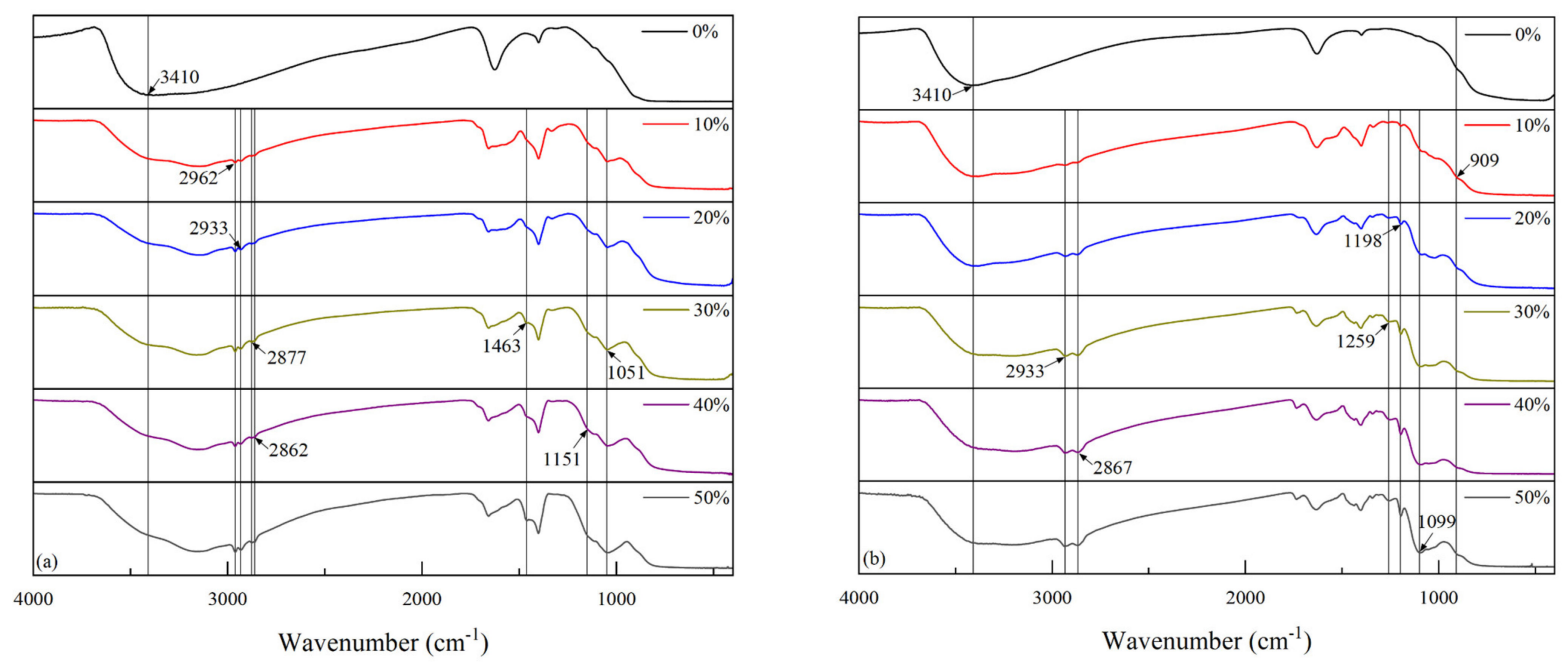
Infrared absorption spectrum of nano-TiO_2_ modified by (**a**) NDZ-201 and (**b**) KH-560.

FT-IR spectroscopy was used to characterize the chemical bonding changes before and after the modification of the two coupling agents with different proportions, and the IR spectral analysis of the NDZ-201-modified nano-TiO_2_ with different modification ratios is shown in Figure 3a. With an increase in the ratio, we observed a gradual increase in the absorption peaks at 2962, 2933, 2877, 2862, 1463, 1151, and 1051 cm^−1^ for the NDZ-201-modified TiO_2_ nanoparticles. Combined with related reports [24], the peaks at 2962, 2933, 2877, and 2862 cm^−1^ were identified as the C–H stretching vibration peaks; 1463 cm^−1^ as the C–H bending vibration peak; 1151 cm^−1^ as the P = O characteristic peak; and 1051 cm^−1^ as the P–O–P characteristic peak. In addition, the stretching vibration peak corresponding to –OH at 3410 cm^−1^ first weakened and remained roughly unchanged with an increase in the modification ratio, indicating that the –OH groups on the nano-TiO_2_ surface were initially continuously consumed during the reaction, and then they reacted completely with the coupling agent molecules when the ratio was near 30 wt%.

The IR spectra analysis of the KH-560-modified nano-TiO_2_ with different modification ratios is shown in Figure 3b. With an increase in the ratio, we observed a gradual increase in the absorption peaks at 2933, 2867, 1259, 1198, 1099, and 909 cm^−1^ for the KH-560-modified TiO_2_ nanoparticles. Combined with related reports [25], the peaks at 2933 cm^−1^ and 2867 cm^−1^ were identified as the C–H stretching vibration peaks; 1259 cm^−1^ as the asymmetric stretching vibration peak of the epoxy group; 1198 cm^−1^ as the C–O–C stretching vibration peak; 1099 cm^−1^ as the Si–O–Si stretching vibration peak; and 909 cm^−1^ as the symmetric stretching vibration peak of the epoxy group. The absorption peak at 3410 cm^−1^ did not change significantly with an increase in the ratio, which was related to the silanol produced by the hydrolysis of KH-560. The intensity of this absorption peak was approximately unchanged when the ratio was near 30 wt%, indicating that the –OH groups on the surface of the TiO_2_ had reacted completely with the coupling agent molecules.

**Figure 4:**
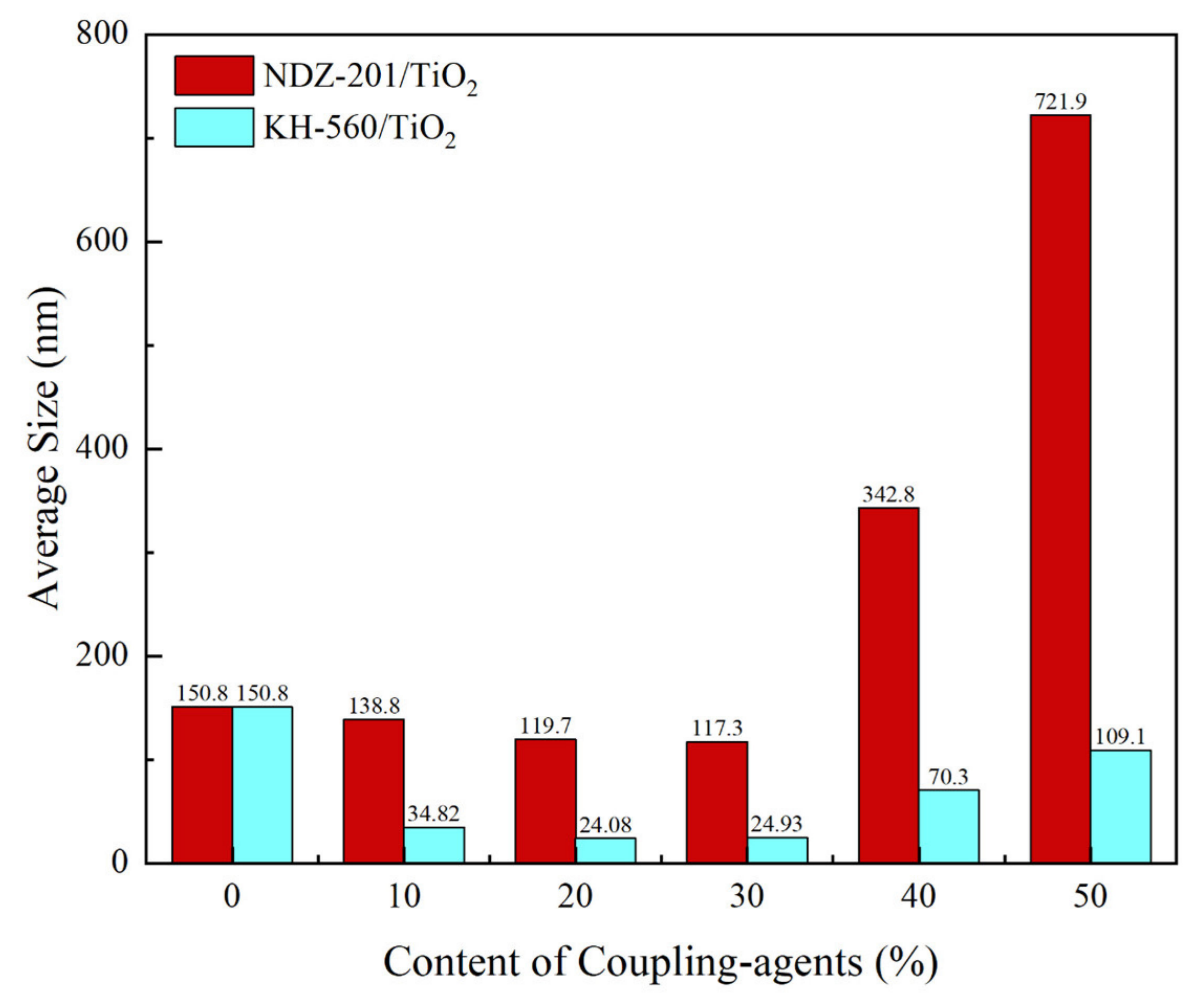
Average particle size of nano-TiO_2_ with different coupling agents and modified ratios.

The average particle size of nano-TiO_2_ modified by the two coupling agents at different modification ratios was tested by a Malvern laser particle size analyzer, and the results are shown in Figure 4. From the overall effect of coupling agents on the average particle size, the average particle size of nano-TiO_2_ could be reduced by the modification of the coupling agents within a certain ratio, which meant that the surface energy of nano-TiO_2_ was reduced during the surface modification process, implying that the surface modification of nano-TiO_2_ by the coupling agents with an appropriate modification ratio could inhibit the tendency of the nanoparticles to agglomerate. However, the average particle size of modified TiO_2_ increased with an increase in the modification ratio when the ratio was greater than a certain value, along with a greater absolute increase in the NDZ-201 group. The reason was different for each coupling agent. The NDZ-201 molecule had three dioctyl pyrophosphate acyloxy groups per molecule, so the intermolecular force of the coupling agent was larger, and it was difficult to disperse the modified TiO_2_ in anhydrous ethanol when the coupling agent quantity exceeded a certain value. The KH-560 molecules could form Si–O–Si bridge bonds between the modified TiO_2_ particles (shown in Figure 1b), which was more significant when the coupling agent was overdosed, leading to an increase in the average particle size. We concluded that KH-560 could inhibit the agglomeration tendency of the TiO_2_ nanoparticles to a greater extent compared to NDZ-201 in an anhydrous ethanol environment. For NDZ-201, the suitable modification ratio was about 30 wt% (relative to nano-TiO_2_), while the ratio was 20 wt% for KH-560.

**Figure 5:**
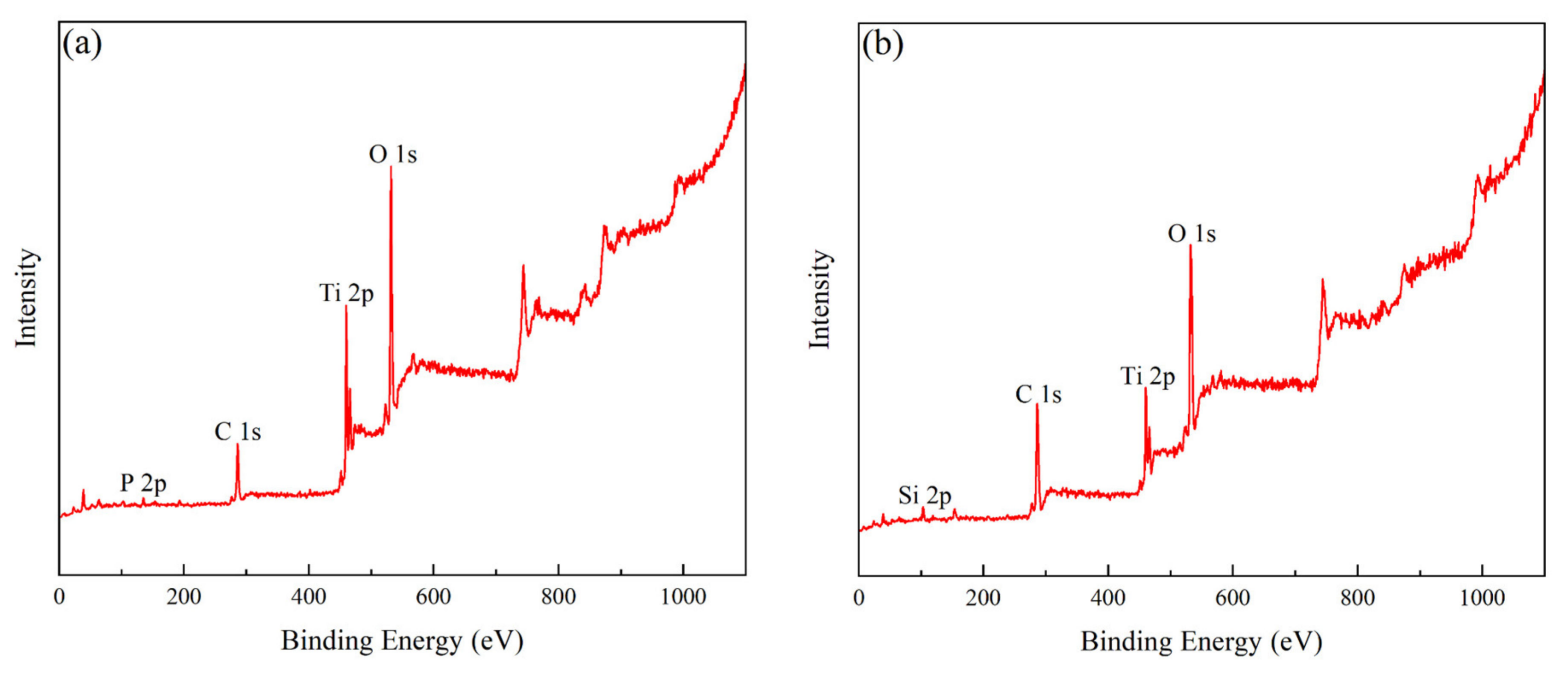
X-ray photoelectron spectra of nano-TiO_2_ modified by (**a**) NDZ-201 and (**b**) KH-560.

The two kinds of modified nano-TiO_2_ particles (for ease of comparison, the modified ratios were all 30 wt%) were characterized by an X-ray photoelectron spectrometer (XPS), and the results are shown in Figure 5. The surface of nano-TiO_2_ modified by NDZ-201 contained P, and the surface modified by KH-560 contained Si, which proved that the two coupling agents were successfully grafted onto the nano-TiO_2_ particle surface. In addition, KH-560 did not contain Ti, so the absorption peak intensity of Ti in Figure 5b was significantly smaller than that in Figure 5a. It was determined that C all came from the two coupling agents themselves. The mass fraction of C in NDZ-201 (C_51_H_112_O_22_P_6_Ti, MW. = 1311.13) was 46.91%, while that in KH-560 (C_9_H_20_O_5_Si, MW. = 236.34) was 45.93%. There was little difference between the number of C atoms in the two coupling agents of equal mass, but the absorption peak intensity of C in Figure 5a was much less than that in Figure 5b. The results showed that the adsorption mass of NDZ-201 on the surface of nano-TiO_2_ was less than KH-560 at the modification ratio of 30 wt%. Moreover, the chemical shifts of each element in Figure 5a,b were very close, which showed that the valence state of each element did not change significantly during the modification process.

**Figure 6:**
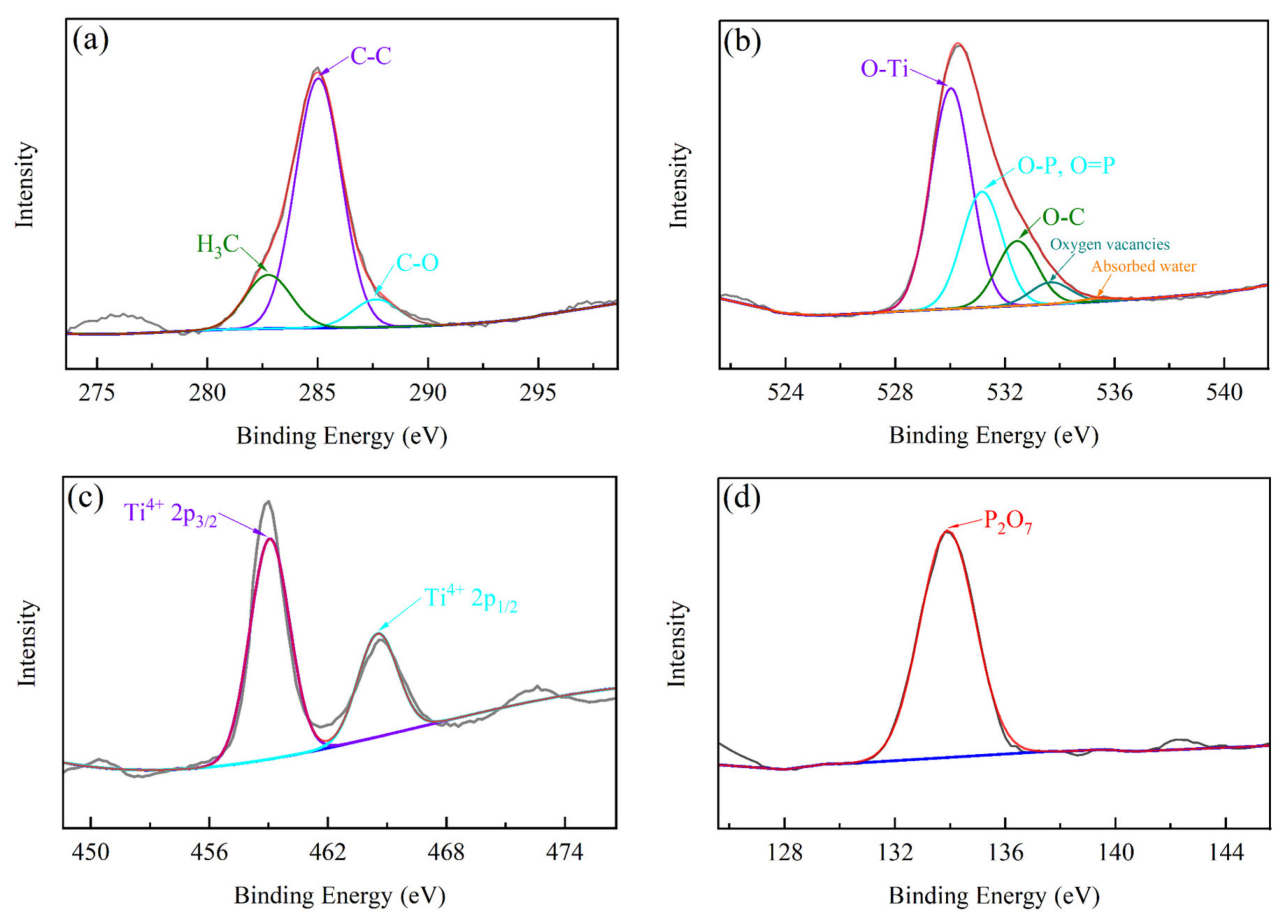
High-resolution XPS spectrum of NDZ-201 modified nano-TiO_2_ at (**a**) C 1s; (**b**) O 1s; (**c**) Ti 2p3; and (**d**) P 2p3.

**Figure 7:**
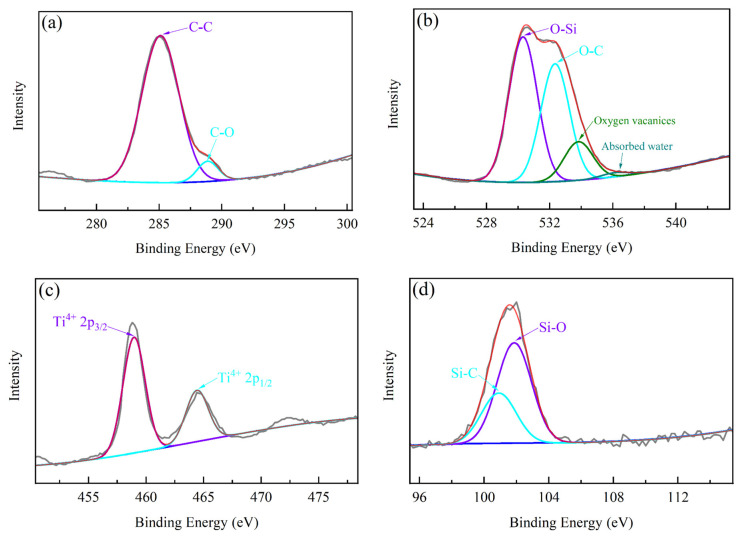
High-resolution XPS spectrum of KH-560 modified nano-TiO_2_ at (**a**) C 1s; (**b**) O 1s; (**c**) Ti 2p3; and (**d**) Si 2p3.

The peaks of the surface elements were fitted by the peak fitting process, according to the conclusions found in the literature [26], and the results are shown in Figure 6 and Figure 7. As shown in Figure 6, the characteristic peak of the –P_2_O_7_ group appeared in the spectrum, which proved that the pyrophosphate structure within the NDZ-201 molecule was not destroyed after the modification reaction. The intensity of the –CH_3_ peak was much smaller than the C–C peak, which indicated that the isopropoxy group in the molecular structure of NDZ-201 no longer existed, and the –CH_3_ group in the structure was only present in the –C_8_H_17_ group after modification. This was because the isopropoxy group reacted with the surface –OH group of the surface of the nanoparticles during the modification process, connecting the surface of the nano-TiO_2_ with NDZ-201. As presented in Figure 7, the XPS spectrum no longer showed the presence of the –CH_3_ peak, which proved that the –OCH_3_ group in KH-560 was completely hydrolyzed after the reaction. In addition, the binding energy of the C–O peak in Figure 7 was larger than in Figure 6, due to the large binding energy of C–O in the epoxy structure of KH-560, indicating that the epoxy groups of KH-560 remained stable during the modification process.

### 3.2. Isothermal Curing Process of the Prepolymers

**Scheme 1:**
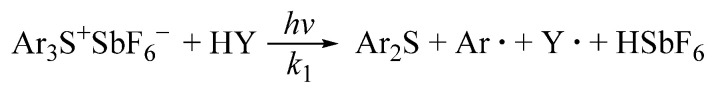
Reaction of the photoinitiator under UV irradiation.

The UV-normal temperature synergistic curing process mechanism of prepolymers can be divided into two stages: the UV irradiation initiation reaction and the normal-temperature curing reaction [27]:

- UV irradiation initiation reaction:

The photoinitiator Ar_3_S^+^SbF_6_^−^ reacted with trace amounts of active hydrogen compounds HY (such as H_2_O) in the system under UV irradiation to produce the super-acid HSbF_6_, as shown in Scheme 1, where *k*_1_ denotes the reaction rate constant in this reaction, and HSbF_6_ is extremely acidic and will easily dissociate the hydrogen ion.

**Scheme 2:**
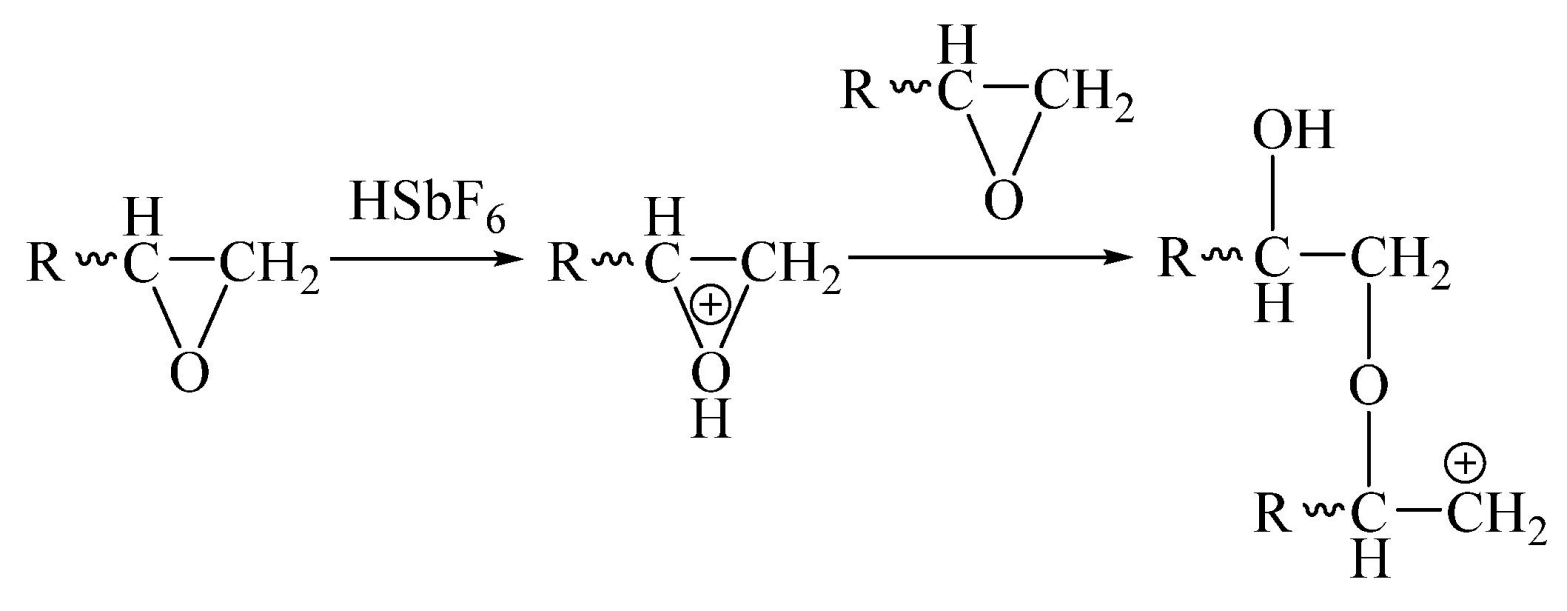
Interconnection of epoxy molecular chains catalyzed by the acid.

As shown in Scheme 2, the positively charged group formed by the dissociated H^+^ combined with the epoxy group and attacked the next epoxy resin macromolecular chain, opening the ring of the epoxy group and connecting the two epoxy resin macromolecules. As a result, a positive charge was transferred to the new epoxy macromolecular chain. This cycle repeated itself and eventually built a large cross-linked network.

Under UV irradiation, the prepolymer was cured according to the cationic polymerization mechanism described above. As a result, the epoxy polymer chains were connected to each other with –CH_2_O–groups, with *k*_1_ as the initiation reaction rate, which mainly depended on the efficiency of the initiator to produce H^+^ by UV irradiation. According to the formation of super-acid HSbF_6_, the concentration of active hydrogen compound *c*_HY_ and irradiation coefficient *K* were the main factors for determining *k*_1_, as shown in Equation (3), where *A* is a constant, $c_{{\mathrm{Ar}}_{3}S^{+}{\mathrm{SbF}}_{6}^{-}}$ is the concentration of triarylsulfonium hexafluoroantimonate salts, and *m* and *n* are defined as the orders of the reaction. It can be seen that nano-TiO_2_ and the coupling agents played a role in the UV initiation reaction; in accordance with Equation (3), nano-TiO_2_ acted as a scattering agent to reduce the irradiation coefficient *K*, resulting in a decrease in *k*_1_ [28]. However, the –OH group on the surface could provide active hydrogen, causing *c*_HY_ to increase and *k*_1_ to rise.

(3) $$k_{1}{=A\cdot c}_{{\mathrm{Ar}}_{3}S^{+}{\mathrm{SbF}}_{6}^{-}}{}^{m}{\cdot c}_{\mathrm{HY}}{}^{n}\cdot K$$

For the two coupling agents NDZ-201 and KH-560, the effect of surface modification on *c*_HY_ had to be determined by comparing their structure with the consumption surface –OH groups during the modification process. Each NDZ-201 molecule contained three pyrophosphate groups, and each pyrophosphate group contained one –OH group, while there were no –OH groups in the molecular structure of KH-560. Therefore, compared to unmodified nano-TiO_2_, modification by NDZ-201 could increase *c*_HY_ and by KH-560 could reduce *c*_HY_, eventually leading to differences in the performance of nano-TiO_2_ modified by different coupling agents in the UV irradiation initiation reaction.

**Scheme 3:**
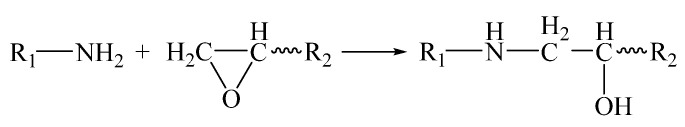
Nucleophilic addition reaction of the primary amino group to the epoxy group.

- Normal-temperature curing reaction:

Triethylenetetramine, as a normal-temperature curing agent, reacted with the epoxy groups through the nucleophilic addition mechanism. Each triethylenetetramine molecule contained four reaction sites, including two primary amine nitrogen atoms and two secondary amine nitrogen atoms. The reaction process of the primary amine nitrogen atom with the epoxy group is shown in Scheme 3.

**Scheme 4:**
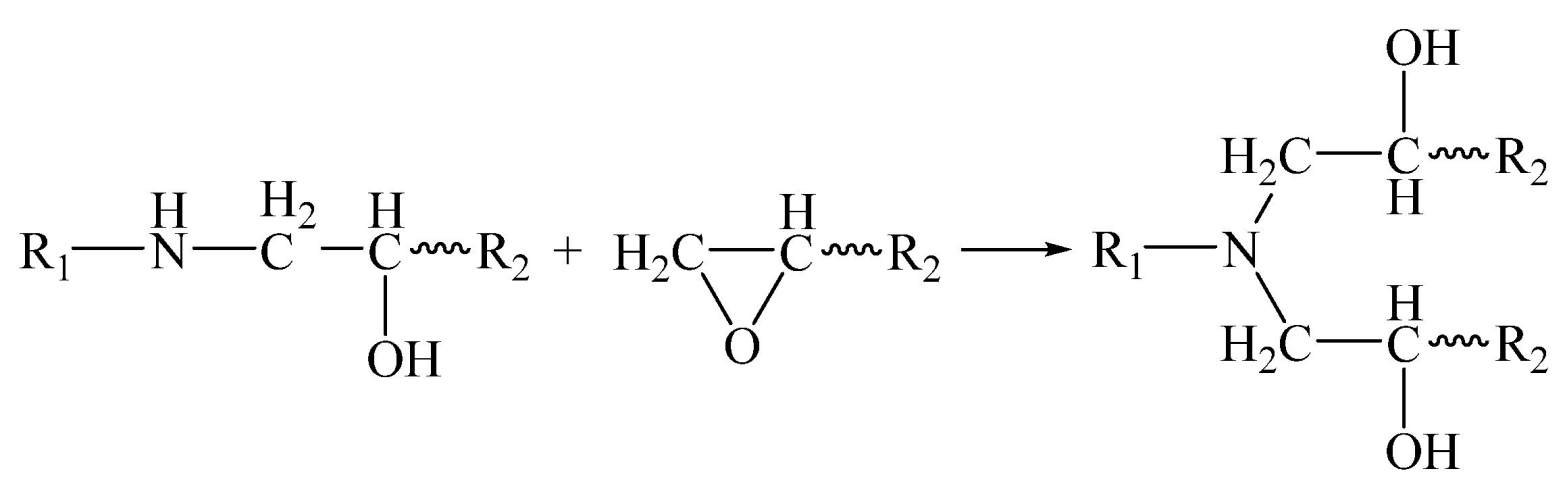
Nucleophilic addition reaction of the secondary amino group to the epoxy group.

After the above reaction, the primary amine nitrogen atom was converted into the secondary amine nitrogen atom, and the reaction process between the secondary amine nitrogen atom and the epoxy group is shown in Scheme 4.

Each triethylenetetramine molecule could connect six epoxy polymer chains together. Turi et al. [29] studied the effect of nanoparticles (such as nano-TiO_2_) on the reaction rate in the heat curing reaction. First, nano-TiO_2_ provided more reaction sites for the curing reaction, and second, the –OH groups on the nano-TiO_2_ surface could catalyze the ring-opening reaction of the epoxy groups. Both points increased the reaction rate of the curing process.

**Figure 8:**
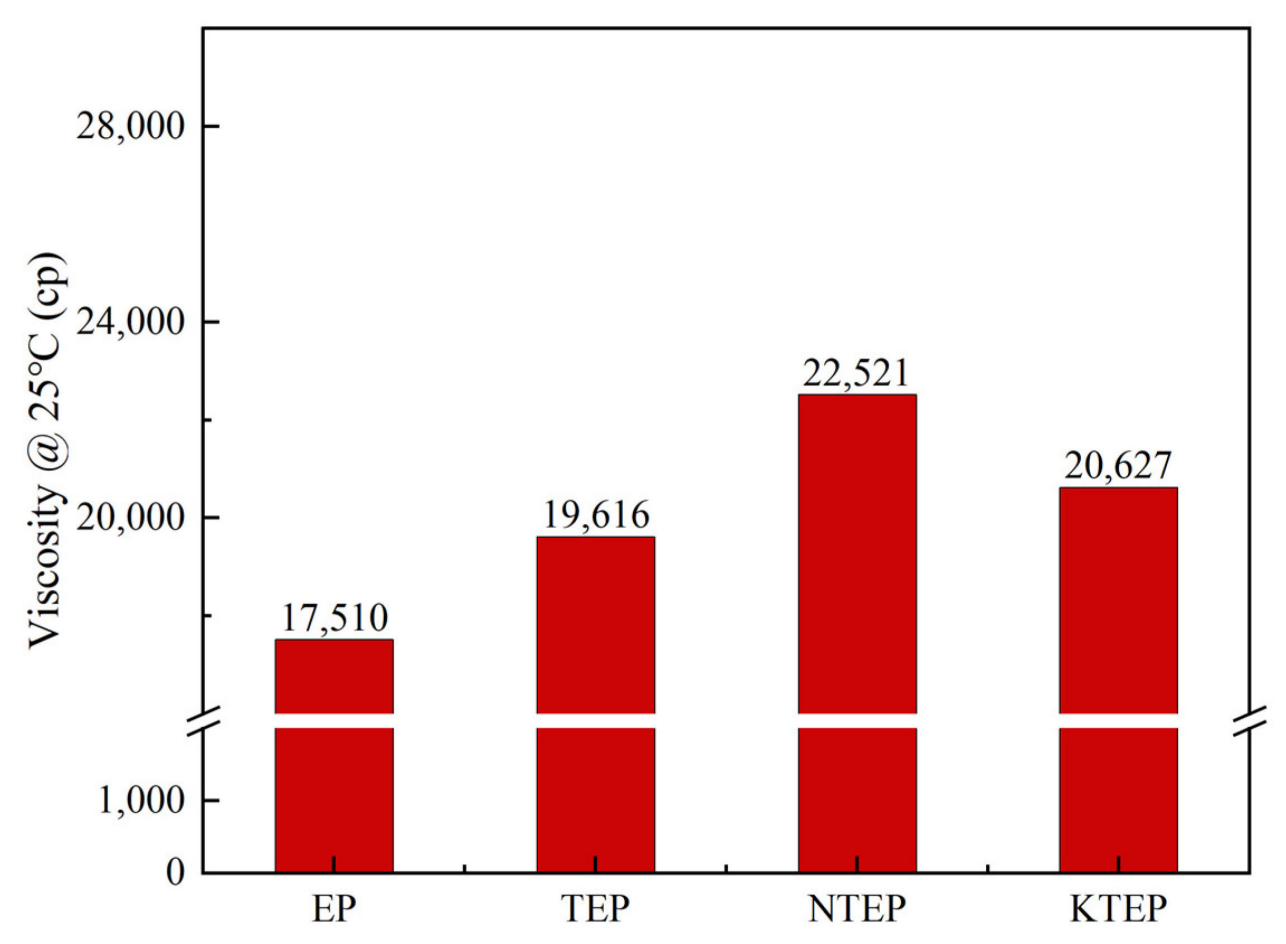
Viscosity of prepolymers at room temperature.

However, the viscosity of the curing system gradually became the dominant factor affecting the curing reaction rate after the middle and later periods of the curing reaction, where the greater the viscosity of the system, the greater the resistance of the polymer chain segment, and the more difficult the curing reaction. The viscosities of the four prepolymer systems, namely EP, TEP, NTEP, and KTEP (for ease of comparison, the modification ratios relative to fillers were all 30 wt%; the ratios of fillers to E-51were all 1 wt%), were measured at room temperature, as shown in Figure 8, and the relative viscosity order of the prepolymers was NTEP > KTEP > TEP > EP. The viscosity of the prepolymer containing nano-TiO_2_ was greater than the pure epoxy resin. Due to the addition of filler particles, the epoxy resin matrix changed from a homogeneous system to a heterogeneous system, where the high surface energy and agglomeration tendency of the nanoparticles further increased the viscosity of the prepolymer. The coupling agents could reduce the viscosity of the system by inhibiting the agglomeration of the nanoparticles and improving the viscosity due to their inherent high intermolecular forces. The NTEP system had the greatest viscosity due to the large tail chain and intermolecular force of the NDZ-201 molecules, which became an important factor that affected the curing rate of the NTEP system.

In the isothermal curing process, at time *t*, the heat flow rate could be defined as *dH*/*dt*, and the current heat of reaction Σ*H_t_* was defined by Equation (4).

(4) $${\Sigma H}_{t}=\int_{0}^{t}\left(dH/dt\right)dt$$

When the reaction was nearly complete, the time was *t*_max_, and the curing degree of the system was *α* = *α*_max_. The heat flow rate *dH*/*dt* of the system conformed to Equation (5).

(5) $$\underset{t\to t_{\max}}{\lim}\frac{d\left(dH/dt\right)}{dt}=0$$

Assuming the curing process was complete at this point, i.e., *α* = 1, the total heat emitted in this reaction Σ*H* could be defined by Equation (6).

(6) $$\Sigma H=\int_{0}^{t_{\max}}\left(dH/dt\right)dt$$

Because *dH*/*dt* was proportional to *dα/dt* (the ring-opening rate of the epoxy group and also the curing reaction rate) in the isothermal curing reaction, the heat of reaction Σ*H*_t_ was proportional to the curing degree *α*, as shown in Equation (7).

(7) $$\alpha =\frac{{\Sigma H}_{t}}{\Sigma H}$$

Subsequently, *dα*/*dt* could be defined by Equation (8).

(8) $$d\alpha /dt=\frac{dH/dt}{\Sigma H}$$

**Figure 9:**
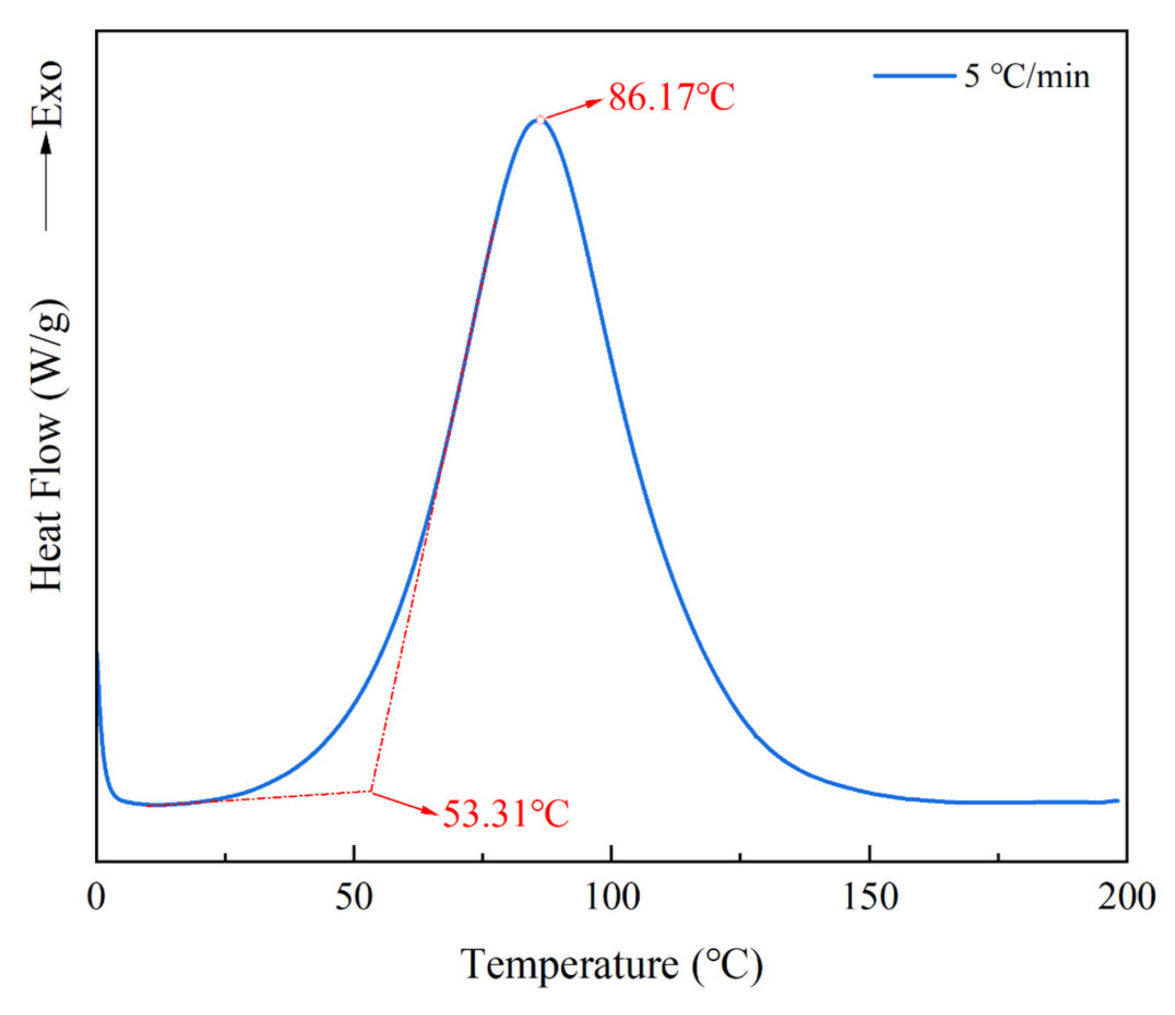
Dynamic DSC curve of EP prepolymer curing.

**Figure 10:**
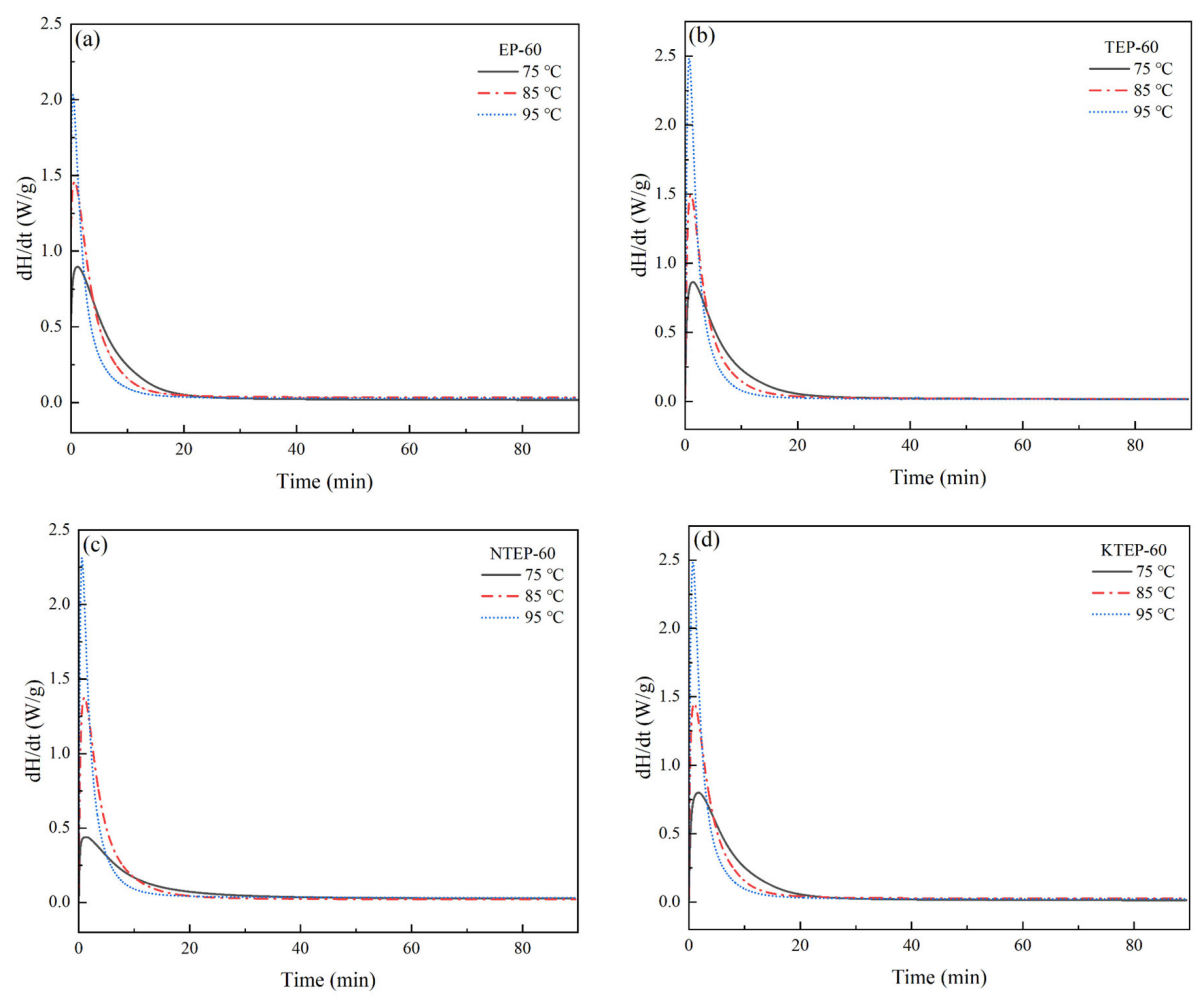
Isothermal DSC curves of (**a**) EP-60; (**b**) TEP-60; (**c**) NTEP-60; and (**d**) KTEP-60 prepolymer at different curing temperatures.

**Figure 11:**
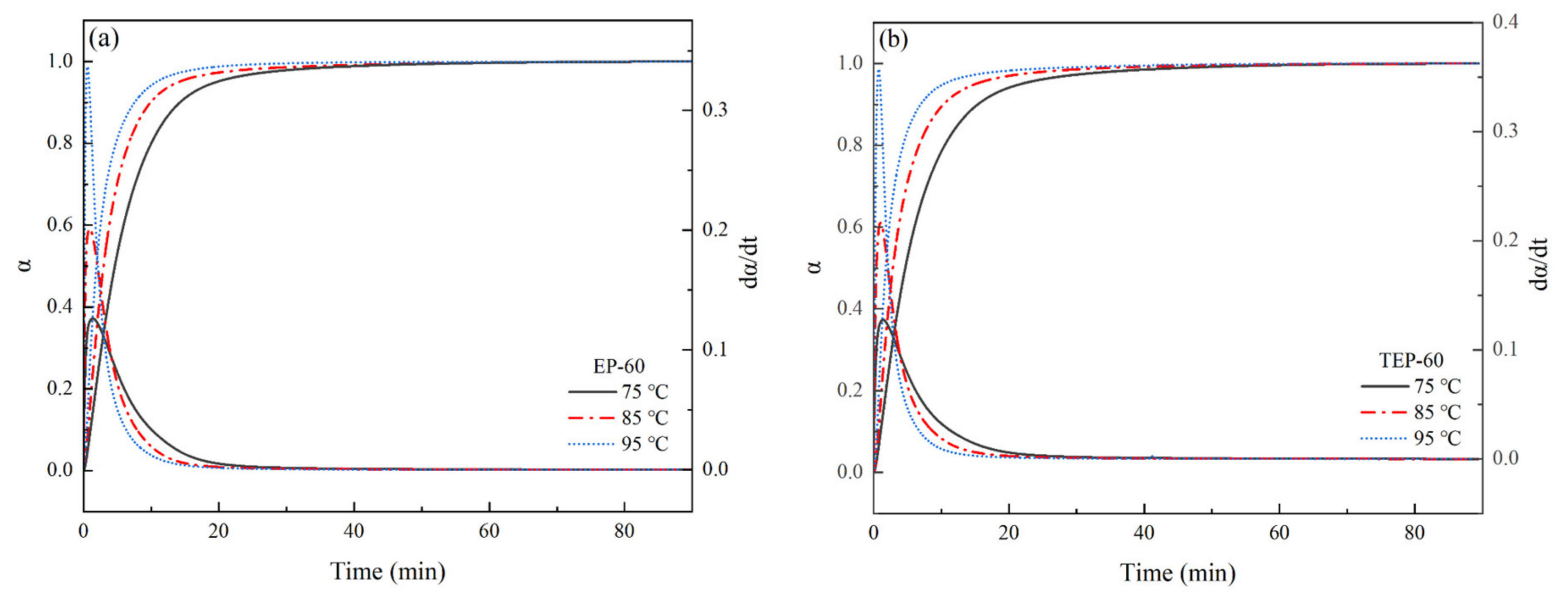

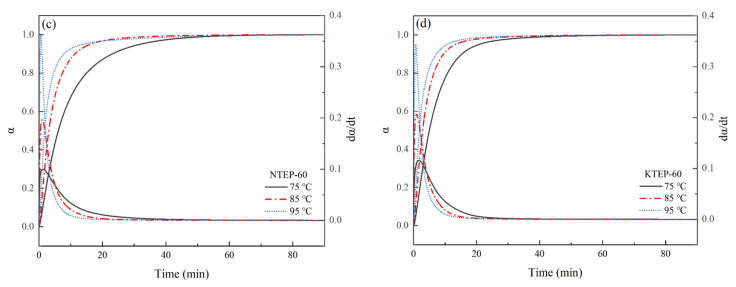
*α-t* and *dα*/*dt-t* curves of (**a**) EP-60; (**b**) TEP-60; (**c**) NTEP-60; and (**d**) KTEP-60 prepolymer isothermal curing systems at different curing temperatures.

The EP system prepolymer was irradiated in the UV light box (the UV irradiation condition in this step was 60 s per cycle), and then we performed dynamic DSC scanning to determine the suitable isothermal curing temperature range. The results are shown in Figure 9. The initial curing temperature *T_i_* of the EP-60 system was 53.31 °C, and the peak temperature *T_p_* was 86.17 °C. The *T_p_* values of the four systems EP-60, TEP-60, NTEP-60, and KTEP-60 were relatively close, considering that the curing conditions of the modified epoxy resin prepolymer were not significantly different from those of the pure epoxy resin prepolymers. We selected 75 °C, 85 °C, and 95 °C as the isothermal curing temperatures of each system, and the isothermal curing curves of the four systems after UV irradiation are shown in Figure 10. The data in Figure 10 were processed according to Equations (7) and (8), and the relationships of *α–t* and *dα*/*dt*–*t* were obtained, respectively, as shown in Figure 11.

**Figure 12:**
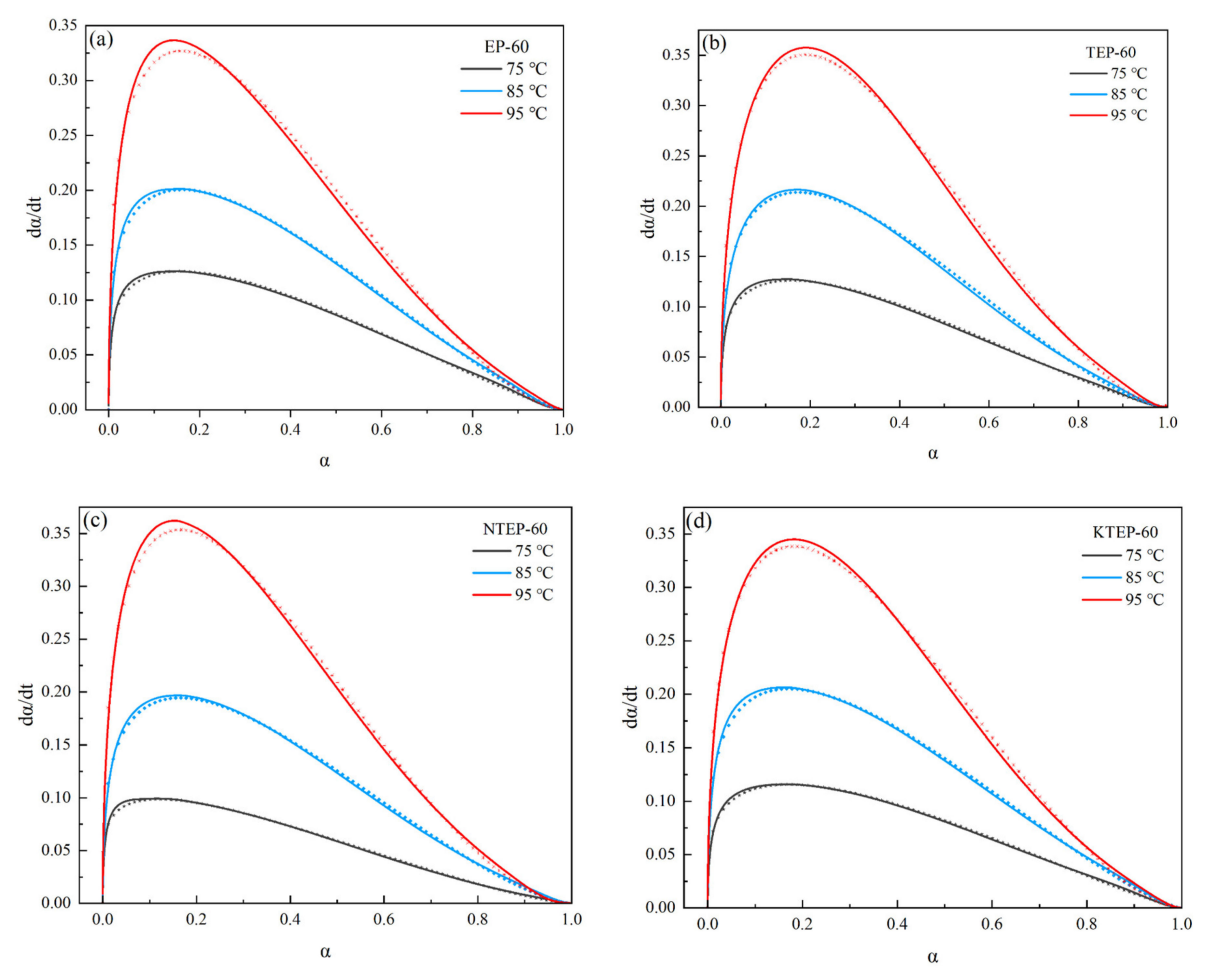
*dα*/*dt-α* experimental results and Kamal model fitting results of (**a**) EP-60; (**b**) TEP-60; (**c**) NTEP-60; and (**d**) KTEP-60 prepolymer isothermal curing systems at different temperatures.

As shown in Figure 10 and Figure 11, the times for each system to reach the maximum curing heat flow rate (*dH*/*dt*)_max_ gradually shifted forward with an increase in isothermal curing temperature, and the times required to reach the maximum curing degree *α*_max_ gradually shortened, where the higher the temperature, the higher the chain segment movement rate, the higher the number of epoxy groups and curing agent molecules that participated in the curing reaction, the more intense the cross-linking reaction during the curing process, and the faster the increase in *α*. With an increase in the curing degree *α*, the viscosity of the system gradually increased, and the movement of the chain segment was hindered. Therefore, the curing rate *dα*/*dt* of the system gradually decreased after the corresponding time of (*dH*/*dt*)_max_. In addition, the corresponding time of (*dH*/*dt*)_max_ was not the time when the normal-temperature curing started, but the period of time after the start of curing, indicating that the relationship between the curing reaction rate *dα*/*dt* and time *t* conformed to the characteristics of the autocatalytic reaction model. The Kamal model is a commonly used model for describing the kinetics of autocatalytic reactions, following the form shown in Equation (9), where *k*_1_ and *k*_2_ denote the rate constants of the reaction, and *m* and *n* are the stages of the reaction. With the assistance of the Kamal model, the difference between the isothermal curing rate and the curing activation energy of each curing system could be discussed in detail, and the influence of nano-TiO_2_ and the coupling agents on the isothermal curing process could be studied. According to the data shown in Figure 10 and Figure 11, the *dα*/*dt*-*α* relationships of EP-60, TEP-60, NTEP-60, and KTEP-60 were fitted by curve nonlinear regression. The curves obtained by experimentation (solid lines in the figure) and the fitted curves (dashed lines in the figure) are shown in Figure 12.

(9) $$\frac{d\alpha }{dt}=\left(k_{1}{+k}_{2}\alpha^{m}\right){\left(1-\alpha \right)}^{n}$$

As shown in Figure 12, the curing reaction rate *dα*/*dt* increased first with an increase in the curing degree *α*, and gradually decreased close to 0 after reaching the maximum, which was in line with the characteristics of an autocatalytic reaction. The fitting results were in high agreement with the experimental results, and the values of the kinetic parameters *k*_1_, *k*_2_, *m,* and *n* are shown in Table 3.

As shown in Table 3, *k*_1_ and *k*_2_ for each system increased significantly with an increase in the reaction temperature, where the higher the temperature, the stronger the chain movement; the greater the number of epoxy groups, initiator molecules, and curing agent molecules that participated in the reaction; and the faster the reaction. Equation (10) can easily be obtained from Equation (9).

(10) $$K_{1\;}=\underset{t\to 0}{lim}\frac{d\alpha }{dt}$$

Equation (10) shows that *k*_1_ was the curing rate constant when *t* = 0, which corresponded to the curing reaction rate of the prepolymer after UV irradiation. As shown in Table 3, the distribution of *k*_1_ values in the EP-60 system was generally larger than the other three systems containing nano-TiO_2_, indicating that nano-TiO_2_ had a greater influence on the curing reaction rate as the scattering agent for UV irradiation, compared to the active hydrogen provider, decreasing the curing reaction rate during the initial reaction period. When the key parameter determined the reaction process in the middle and later periods of the autocatalytic reaction [30], the *k*_2_ of each system was much greater than *k*_1_ at the same curing temperature, indicating that the influence of the autocatalytic reaction rate constant (*k*_2_) on the curing system was much greater than the initial reaction rate constant (*k*_1_). In addition, the distribution of *k*_2_ values in the three systems containing nano-TiO_2_ was generally higher than in the EP-60 system. This indicated that nano-TiO_2_ had a greater influence on the curing reaction rate as the reaction site and catalyst for the ring-opening reaction, compared to the viscosity enhancer for the epoxy prepolymer, increasing the curing reaction rate more significantly in the middle and later periods of the curing reaction. The distribution of the *k*_2_ values of the TEP-60 system was generally higher than in the two systems containing coupling agents, indicating that the presence of coupling agents had a negative impact on the curing reaction rate of the nano-TiO_2_-containing system (the above discussion is based on the isothermal curing reaction temperature in the range of 75–95 °C).

The rate constants *k*_1_ and *k*_2_ of the isothermal curing reaction were affected by temperature and could be described by the Arrhenius equation, as shown in Equation (11), where *A* is a constant, *E_a_* is the activation energy of the reaction, *R* = 8.314 J/(mol∙K) is the molar gas constant, and *T* is the reaction temperature (measured in K). We took the logarithm of both sides of Equation (11) to obtain Equation (12).

(11) $${k=Ae}^{-\frac{E_{a}}{RT}}$$

(12) $$ln\;k=ln\;A-\frac{E_{a}}{R}\frac{1}{T}$$

**Figure 13:**
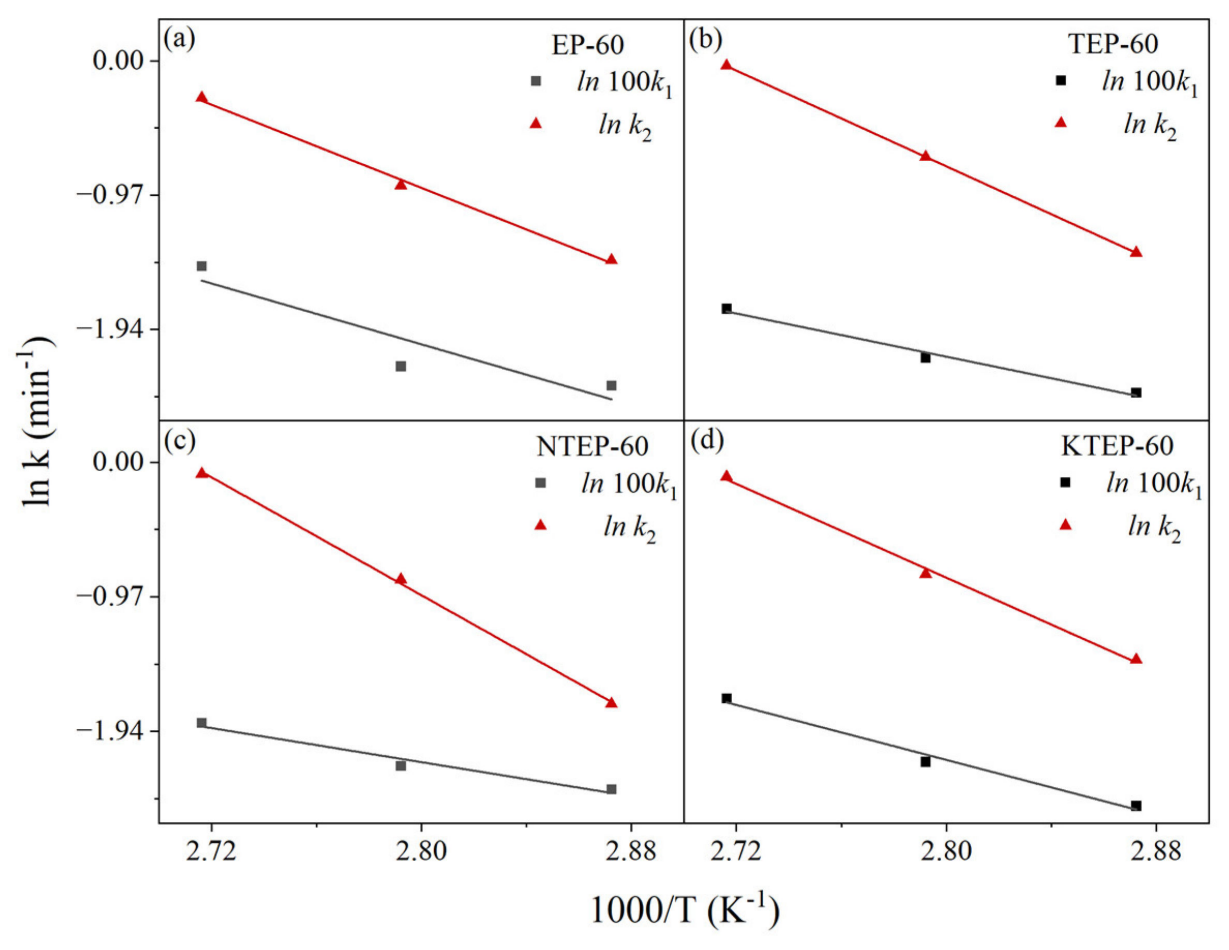
ln *k*-1/*T* curves and fitting results of (**a**) EP-60; (**b**) TEP-60; (**c**) NTEP-60; and (**d**) KTEP-60 curing systems.

According to Equation (12), the activation energies *E_a_*_1_ and *E_a_*_2_ corresponding to the reaction rate constants *k*_1_ and *k*_2_ were obtained by plotting the ln *k*-1/*T* relationship and performing linear regression fitting by OriginPro 2021b, and the results are shown in Figure 13 and Table 4. Similar to the definitions of *k*_1_ and *k*_2_ in the curing reaction, the size of *E_a_*_1_ reflected the height of the reaction energy barrier of the curing system after UV irradiation, and the size of *E_a_*_2_ reflected the height of the reaction energy barrier of the curing system in the middle and later periods of the reaction. The *E_a_*_1_ values of the TEP-60, NTEP-60, and KTEP-60 systems were smaller than the EP-60 system, indicating that the addition of nano-TiO_2_ reduced the energy barrier of the curing system after UV irradiation because the presence of nano-TiO_2_ increased the number of active hydrogen atoms in the system. The *E_a_*_1_ value of the NTEP-60 system was smaller than TEP-60, and that of the KTEP-60 system was greater than TEP-60, due to the inconsistency in the number of active hydrogen atoms of the two coupling agents. Compared to EP-60, the *E_a_*_2_ values of TEP-60, NTEP-60, and KTEP-60 all increased to varying degrees. The order of *E_a_*_2_ values for the four systems was consistent with the order of relative viscosity of the systems in Figure 8, indicating that the *E_a_*_2_ for each system was mainly related to the viscosity of the prepolymer. Based on the above results, nano-TiO_2_ and the coupling agents reduced the energy barrier of the curing reaction of the system after UV irradiation and improved the energy barrier in the middle and later periods of the curing reaction. Therefore, to improve the curing efficiency in the actual production process, the curing temperatures of the epoxy resin prepolymers containing nano-TiO_2_ and the coupling agents were slightly higher than the pure epoxy resin prepolymers.

### 3.3. Characterization of the TiO_2_/Epoxy Resin Composites

**Figure 14:**
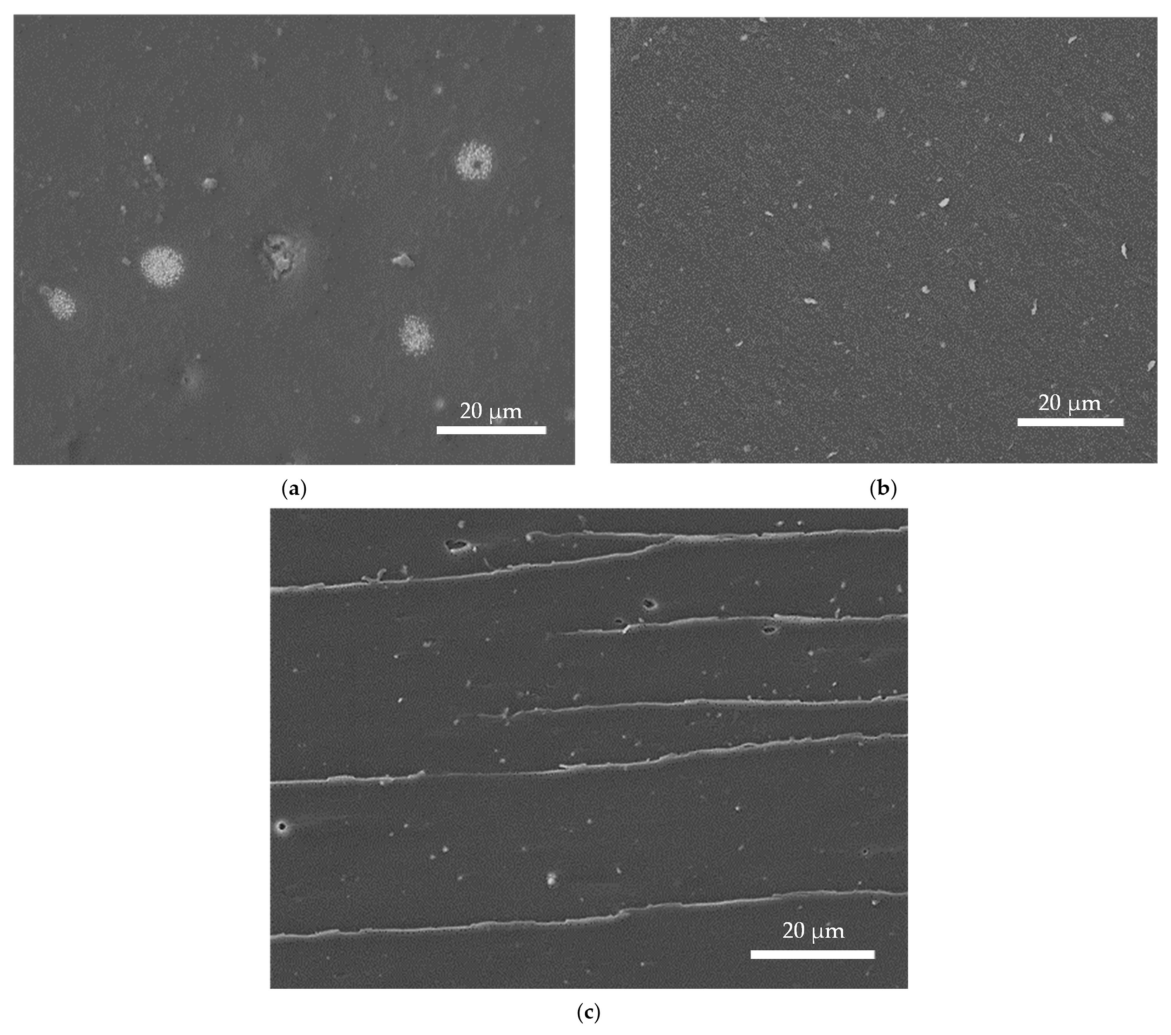
Surface morphologies of (**a**) TEP-60; (**b**) NTEP-60; and (**c**) KTEP-60.

To study the influence of different components on the microstructure of the composites, the fracture surfaces of the three composite materials TEP-60, NTEP-60, and KTEP-60 were observed by scanning electron microscopy (from the perspective of fixed irradiation time as a variable and ensuring the success rate of material preparation, the UV irradiation time of each composite material was 60 s per cycle), and the results are shown in Figure 14.

The white dots on the surfaces were nano-TiO_2_. We found that the phenomenon of particle agglomeration in TEP-60 was significantly more serious, with more even particle distributions in NTEP-60 and KTEP-60. According to the crack nailing theory [31], the solid rigid nanoparticles played the role of nailing points, increasing the energy required for crack propagation during the crack propagation process inside the composite, resulting in the improved mechanical properties of the materials. The stress of crack propagation mainly depended on the particle size and the distance between the particles inside the composite. Compared to the evenly dispersed nano-TiO_2_ particles, agglomerated nano-TiO_2_ particles showed larger particle cluster spacing, which was more conducive to crack propagation. As a result, the crack propagation resistance of the TEP-60 composite material was poor.

**Figure 15:**
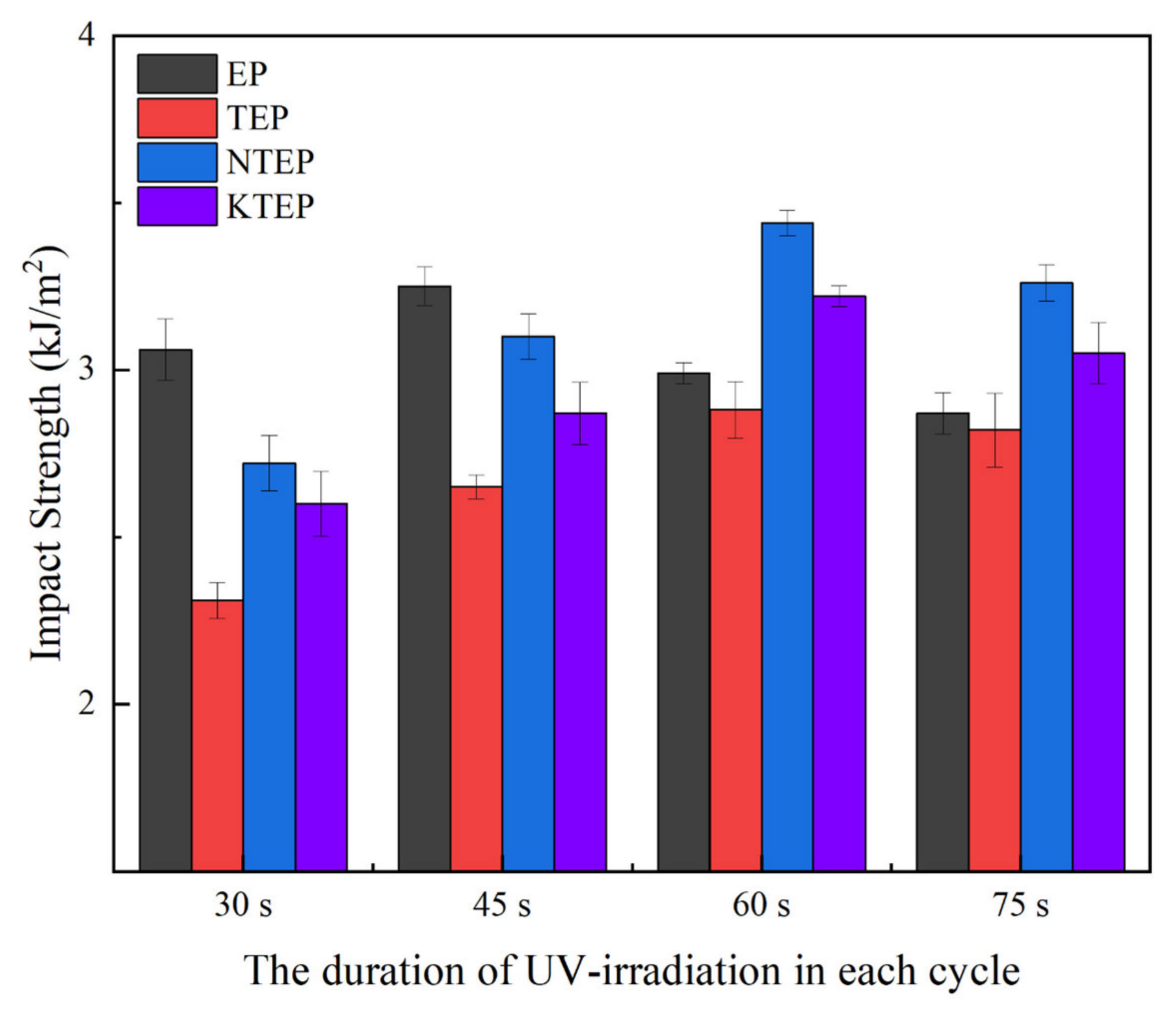
Impact strength of composites in different curing reaction conditions.

**Figure 16:**
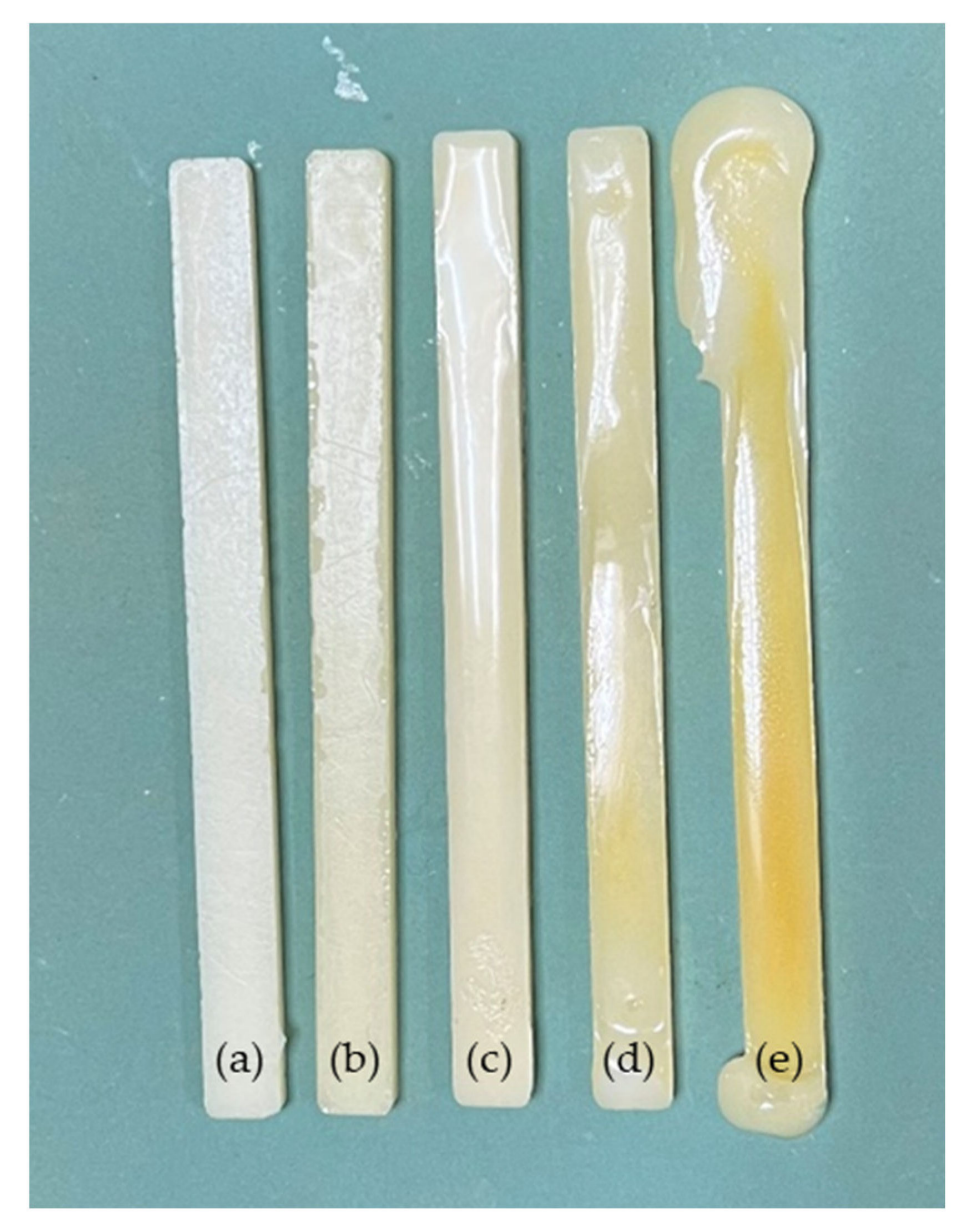
Surface color comparison of (**a**) TEP-0; (**b**) TEP-30; (**c**) TEP-45; (**d**) TEP-60; and (**e**) TEP-75.

Figure 15 shows the impact strengths of the four systems of the composite materials made under different UV irradiation conditions. The impact strength of each composite material system first increased and then decreased with an increase in irradiation time. There was a small number of cross-linking points in the curing system with insufficient UV irradiation, and the cross-linking degree of the sample was low after curing, resulting in poor toughness of the material, which manifested as low impact strength. With an increase in irradiation time, the degree of the yellowing of the cured composites increased, as shown in Figure 16. In the TEP-75 sample, the bisphenol A structure of the material was oxidized, producing carbonyl groups, and the free triethylenetetramine molecules (functioning as the curing agent originally) were directly polymerized with the epoxy resin. The polymer chains were partially damaged in this sample, and as a result, the curing efficiency of the curing agent decreased. In addition, the curing reaction of the photoinitiator was too fast with an increase in irradiation time, and the heat accumulation of the system increased significantly, which was not conducive to the formation of a regular internal structure in the composite material.

**Figure 17:**
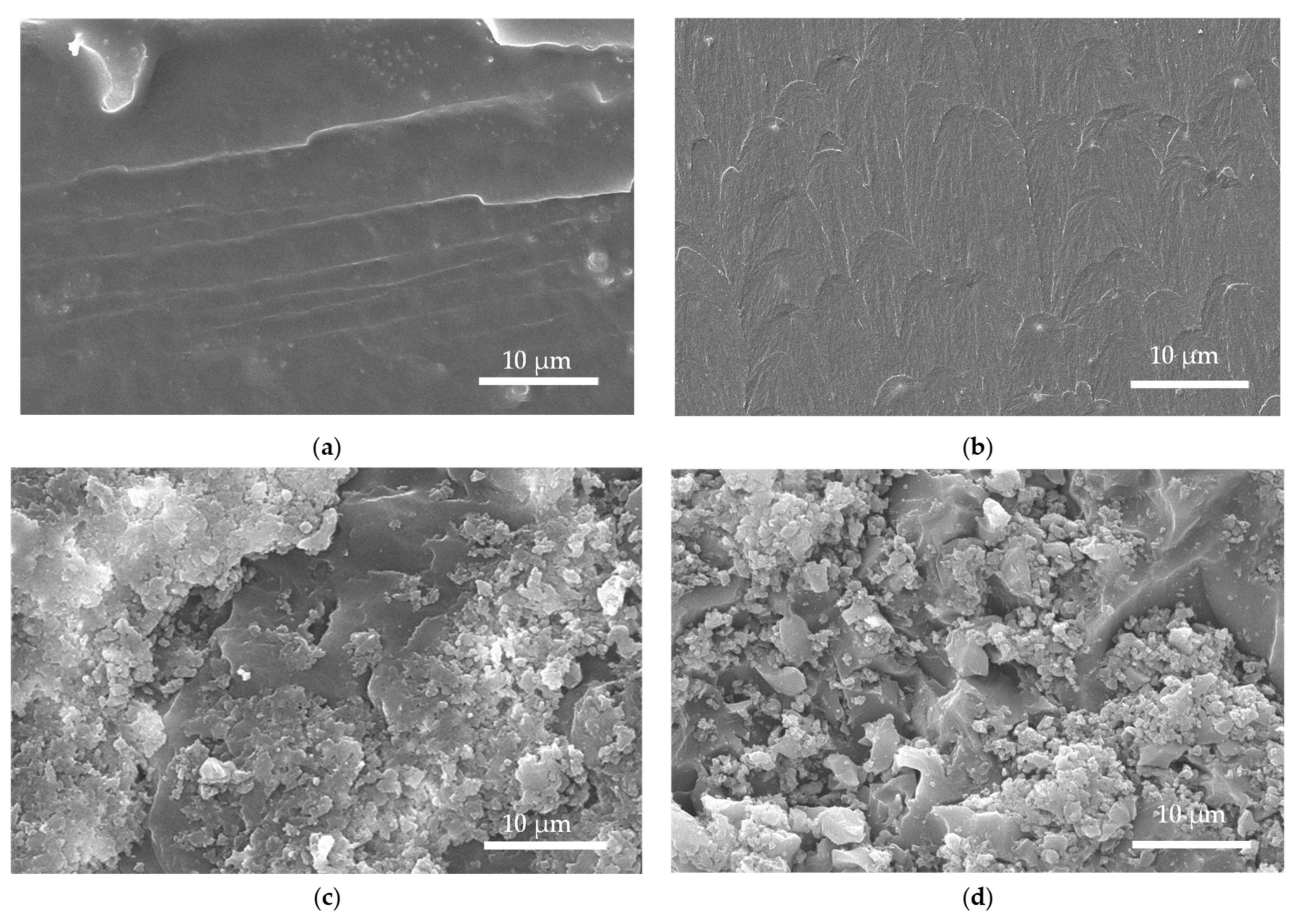
Morphology of the impact fracture surface of (**a**) EP-60; (**b**) TEP-60; (**c**) NTEP-60; and (**d**) KTEP-60.

In the EP system, EP-45 showed the greatest property impact, while the systems with nano-TiO_2_, X-60 (X = TEP, NTEP, and KTEP), had the largest impact strength. The maximum impact strength of the system containing nano-TiO_2_ corresponded to 1/3 more irradiation time than the EP system, because nano-TiO_2_ in the composite matrix decreased the utilization efficiency of the photo-initiator for UV irradiation as a scattering agent. The impact strength of the TEP system was significantly lower than the other systems. As shown in Figure 14, the average particle size of nano-TiO_2_ in TEP was relatively larger, with an obvious agglomeration of the particles in the matrix. The agglomeration of particles not only weakened the resistance to crack propagation of the composites but also reduced the number of reaction sites in the curing system, resulting in a decline in the cross-linking degree of the system and an increase in the stress concentration degree within the material when impacted. Compared to the TEP system, the average particle size of the nanoparticles in the systems containing coupling agents was smaller, with better dispersion of particles in the matrix, so the composite had a larger cross-linking degree and higher impact strength. The impact strength of the NTEP system was slightly higher than the KTEP system, due to the different molecular structures between the two coupling agents. The lipophilic end of the KH-560 molecule was the epoxy group, which was relatively rigid, while the lipophilic end of the NDZ-201 molecule was the –C_8_H_17_ group, which was relatively flexible. The rigid groups exhibited a poor ability to disperse the impact energy through chain segment motion compared to the flexible groups. Figure 17 shows the morphologies of the impact fracture surfaces of the X-60 (X = EP, TEP, NTEP, and KTEP) composite splines, where the fracture surfaces of EP-60 and TEP-60 were relatively smooth, reflecting obvious brittle fracture characteristics.

**Figure 18:**
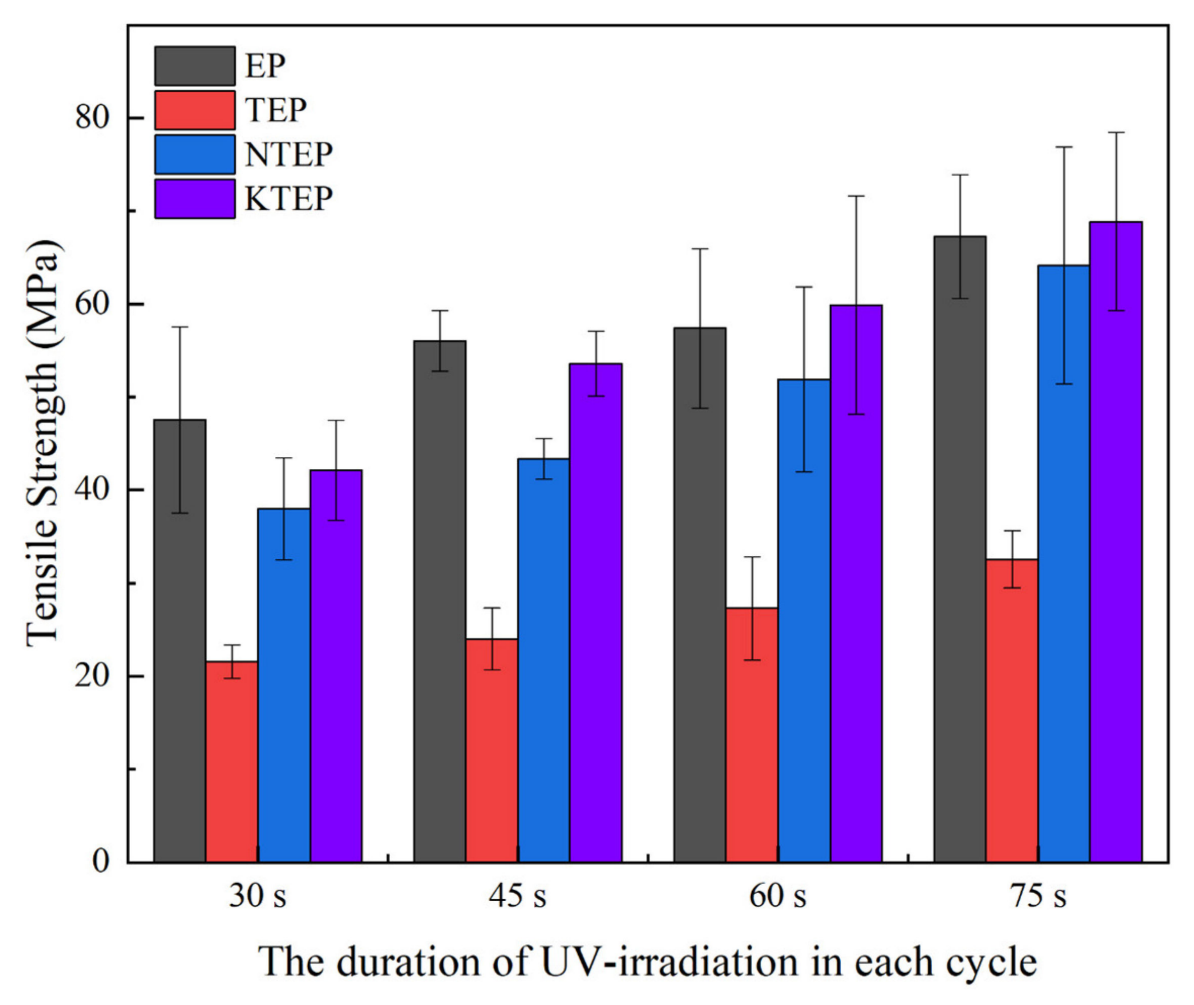
Tensile strength of composites in different curing reaction conditions.

**Figure 19:**
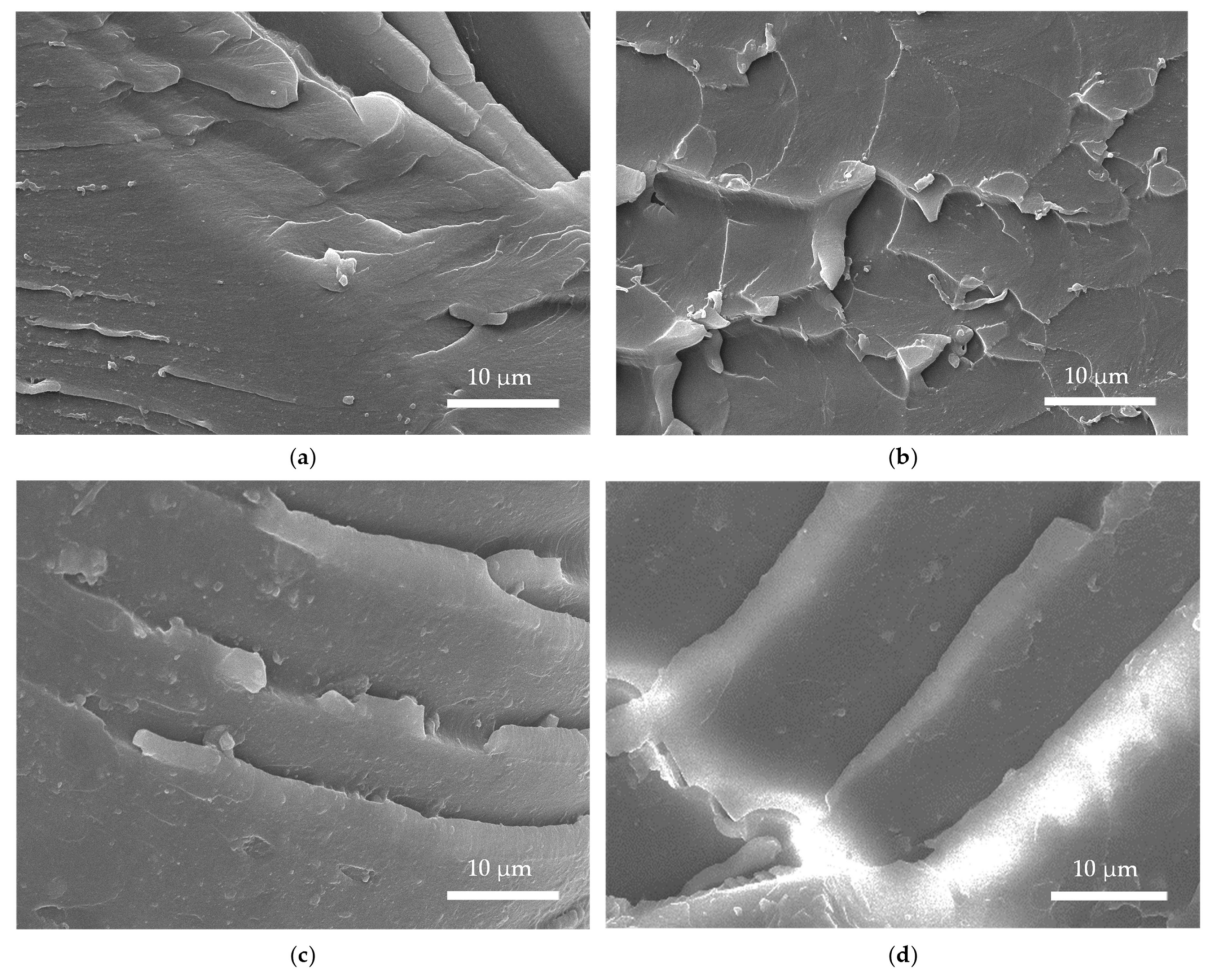
Morphology of the tensile fracture surface of (**a**) EP-75; (**b**) TEP-75; (**c**) NTEP-75; and (**d**) KTEP-75.

Meanwhile, the fracture surfaces of NTEP-60 and KTEP-60 were much rougher, reflecting certain ductile fracture characteristics and greater fracture energy compared to EP-60 and TEP-60. As indicated by the discussion in this section, NDZ-201 imparted more toughness on the composites than KH-560, and among all the composites, NTEP-60 had the highest impact strength. Figure 18 shows the tensile strengths (breaking strengths) of the composite materials composed of the four systems under different UV irradiation conditions. The tensile strengths of the composites of each system increased with an increase in the irradiation time, where the longer the irradiation time, the higher the proportion of cross-links among the epoxy resin polymer chains in the composites. The polymer chains were mainly connected by –CH_2_O–bonds in the UV irradiation reaction, which had a stronger rigidity, while the polymer chains were mainly connected by –CH_2_–N(R)–CH_2_– in the normal-temperature curing reaction, which had a weaker rigidity. Therefore, the longer the irradiation time, the greater the rigidity of the composite material. Due to its low cross-linking degree, the tensile strength of the TEP system was significantly lower than the other systems. With an increase in irradiation time, the leading amplitude in tensile strength of the EP system was gradually reduced or even surpassed the system containing coupling agents, due to the increase in irradiation time, which caused damage to the polymer chain, as it improved the rigidity of the connection between the polymer chains. Due to the scattering effect of nano-TiO_2_ under UV irradiation, the systems containing nano-TiO_2_ were more resistant to excessive irradiation, indicating that the degree of damage among these polymer chains was smaller. Due to the smaller particle size of KH-560-modified nano-TiO_2_, the KTEP curing system had more reaction sites and a greater cross-linking degree. Therefore, the tensile strength of the KTEP system was greater than the NTEP system. In addition, the KH-560 molecule contained an epoxy group, which was relatively rigid and improved the ability of the polymer chain to resist traction. Figure 19 shows the morphologies of the tensile fracture surfaces of the X-75 (X = EP, TEP, NTEP, and KTEP) composite test splines, where each fracture surface showed obvious brittle fracture characteristics. Compared to the fracture surface of TEP-75, the cracks of the other three surfaces were deeper, and the corresponding fracture energies were greater. As indicated by the discussion in this section, KH-560 imparted more rigidity on the composites than NDZ-201, and among all the composites, KTEP-75 had the highest tensile strength.

**Figure 20:**
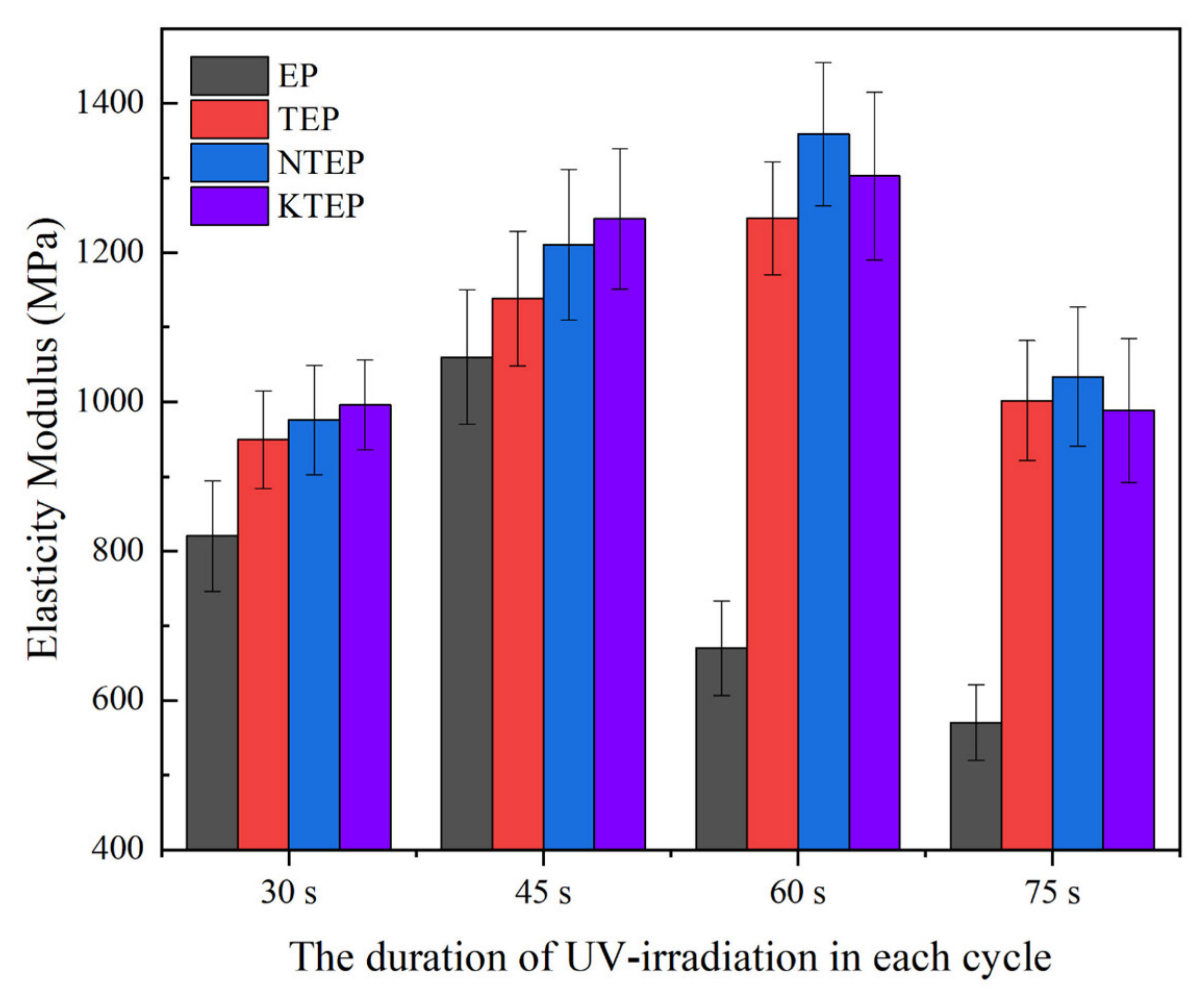
Elastic modulus of composites in different curing reaction conditions.

Figure 20 shows the elastic modulus values of the composite materials made of the four systems under different UV irradiation conditions, where the elastic modulus of each composite material showed the same trend as impact strength. In addition, the elastic modulus trend of each system also showed the following characteristics. In the EP system, the elastic modulus values of EP-60 and EP-75 changed more significantly than EP-45, compared to the previous sample, which was still related to the destruction of the polymer chains caused by excessive irradiation. The elastic modulus values of the systems containing nano-TiO_2_ were significantly greater than the EP system because the –OH groups on the surface of nano-TiO_2_ particles could form hydrogen bonds with the oxygen atoms of the epoxy resin polymer chains, strengthening the forces between the polymer chains and increasing the energy required for polymer chain sliding. The elastic modulus of the systems containing the coupling agents was greater than the TEP system, indicating that the coupling agent could strengthen the ligation effect of nano-TiO_2_ as the linker between the polymer chains. In addition, A and B showed convergence in the elastic modulus test results.

**Figure 21:**
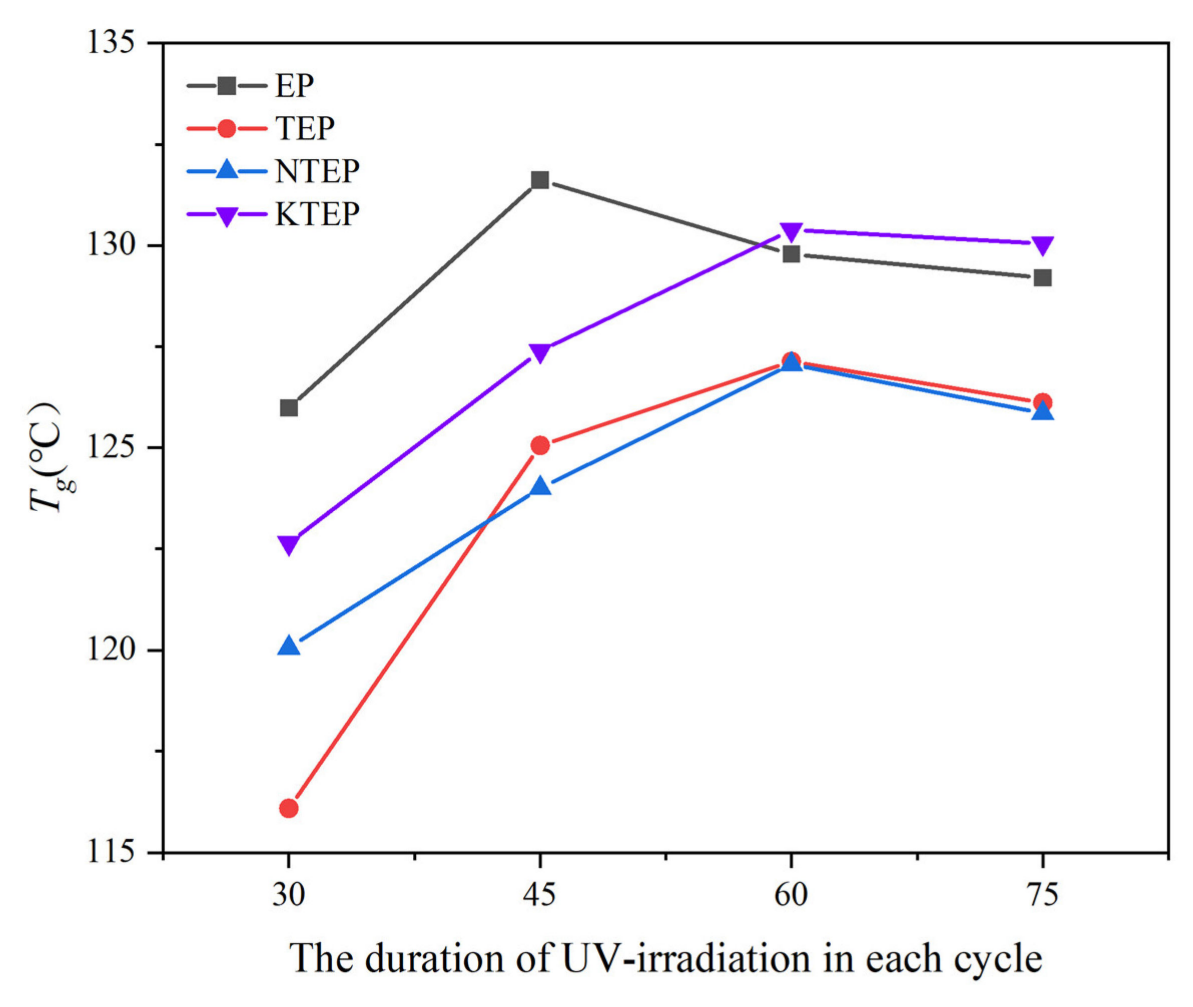
Glass transition temperature of composites obtained after curing of different systems.

The glass transition temperatures (*T_g_*) of the EP, TEP, NTEP, and KTEP composites are shown in Figure 21, indicating that EP-45 had the largest *T_g_* in the EP system, while X-60 (X = TEP, NTEP, and KTEP) had the largest *T_g_* in the corresponding system. These results were similar to the previous impact strength results, which were still related to the scattering effect of nano-TiO_2_ under UV irradiation. The *T_g_* for each system first increased and then decreased with an increase in irradiation time, which was also similar to the trend of the cross-linking density of each system. When compared to each other, the *T_g_* of the TEP system was smaller than the EP system and had a lower cross-linking degree. Preghenella et al. [32] noted that this phenomenon was related to the large viscosity of the prepolymer of the TEP system. In addition, Sun et al. [33] showed that nanoparticles played a plasticizing role in the epoxy resin matrix, which increased the free volume of the system and decreased *T_g_*. The value of the *T_g_* of the NTEP system was similar to the TEP system because NDZ-201 was connected to the epoxy resin matrix with a flexible alkyl tail chain, –C_8_H_17_, which had less effect on *T_g_*. Compared to the TEP and NTEP systems, the *T_g_* of the KTEP system was significantly larger for several reasons. The KH-560-modified nano-TiO_2_ had a small particle size and could easily disperse, which was conducive to increasing the cross-linking density of the system. KH-560 could also easily form Si–O–Si bonds during the reaction, resulting in an increase in the cross-linking degree of the system. The epoxy group at the tail end of the KH-560 molecule was rigid, which restricted polymer chain activity.

**Figure 22:**
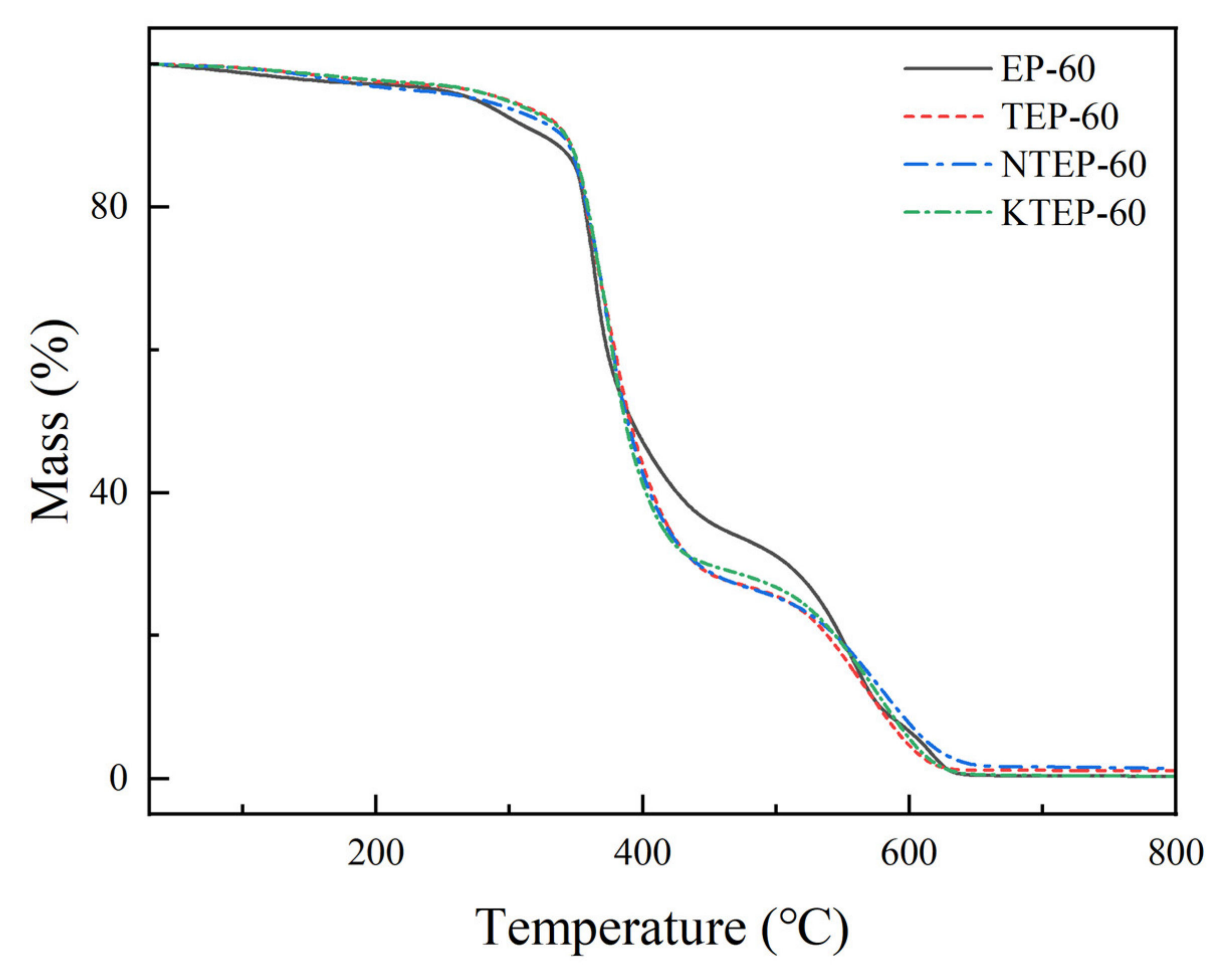
Thermogravimetric curves of each sample.

To study the effects of different components on the heat resistance of the composites, EP-60, TEP-60, NTEP-60, and KTEP-60 were assessed by thermogravimetry. The thermogravimetric curve of each sample is shown in Figure 22, with the thermal decomposition temperature of each sample shown in Table 5. In Table 5, *T*_5%_ refers to the initial decomposition temperature, *T*_30%_ refers to the temperature corresponding to the loss of 30% of the sample mass, and *T_m_* refers to the temperature corresponding to the maximum rate of thermal decomposition. In general, the thermal decomposition of each sample mainly consisted of two steps: the carbonization of each composite material and the combustion of the residual carbon of each system. The *T*_5%_ and *T*_30%_ values of the composites containing nano-TiO_2_ were higher than EP-60, which indicated that nano-TiO_2_ could improve the heat resistance of the composite to a certain temperature range due to its high thermal stability and strong binding force with the epoxy resin polymer chain. This strong binding made the carbon layer denser after combustion, which could hinder the transfer of combustion heat and the infiltration of oxygen. Compared to TEP-60 and KTEP-60, the *T*_5%_ of NTEP-60 was significantly lower because the pyrophosphate groups in the molecular structure of NDZ-201 could easily decompose when heated. The *T_m_* of the composites containing coupling agents improved relative to TEP-60 because of the smaller particle size of the modified nano-TiO_2_, which increased the cross-linking degree of the material.

## 4. Conclusions

In this study, two coupling agents, NDZ-201 and KH-560, were successfully used for the surface modification of nano-TiO_2_ by the solvothermal method, and the characterization results showed that the average particle size of the nanoparticles decreased by up to 22.2% and 84.0% in the modification ratio range of 0~30 wt%. A series of composite materials was fabricated by mixing nano-TiO_2_ with epoxy resin prepolymers, and a series of composites was created by the UV-normal temperature synergistic curing process. We then studied the isothermal curing process of the UV-initiated prepolymers at different temperatures, and the surface morphology, mechanical properties, *T_g_*, and heat resistance of the composites were characterized and compared. The isothermal curing kinetic analysis results showed that nano-TiO_2_ reduced the rate constant and activation energy of the curing reaction of each system after UV initiation and improved the rate constant and activation energy in the middle and later periods of the curing reaction. Nano-TiO_2_ and the two coupling agents could increase the viscosity of the curing system, while NDZ-201 improved the UV initiation efficiency, and KH-560 reduced the UV initiation efficiency. The surface morphology analysis of the composites showed that coupling agent modification could inhibit the agglomeration tendency of nano-TiO_2_ in the epoxy resin matrix. Mechanical property assessment showed that the elastic modulus and impact strength of the composite materials initially increased and then decreased with an increase in UV irradiation time, while the tensile strength showed a gradual increase. The morphology results of the impact and tensile fracture surfaces showed that the presence of nano-TiO_2_ and the coupling agents improved the fracture energy of the composites, while the *T_g_* results showed that KH-560 had the greatest increase in the *T_g_* of the epoxy matrix composite system. The heat resistance test results showed that nano-TiO_2_ improved the heat resistance of the composite. Compared to the two coupling agents, the impact strength of NTEP-60 was up to 6.8% higher (minimum value; the same below), and the maximum thermal decomposition temperature was 4.88 °C higher. In addition, the tensile strength of KTEP-60 was up to 7.3% higher, and *T_g_* was 3.34 °C higher. This study describes the effect of nano-TiO_2_, NDZ-201, and KH-560 on epoxy resin composite systems from the aspects of the curing process and the mechanical and heat resistance properties of the materials, which provides a reference for the determination of UV curing process parameters of nano-TiO_2_/epoxy composites.

## Figures and Tables

**Table 1:** The minimum and maximum irradiation energy under different irradiation times.

Irradiation Time	Minimum Irradiation Energy/kJ·m^−2^	Maximum Irradiation Energy/kJ·m^−2^
30 s	174	213
45 s	261	320
60 s	348	426
75 s	435	532

**Table 2:** Relationship between each sample code and the corresponding irradiation time per cycle.

System	30 s/Cycle	45 s/Cycle	60 s/Cycle	75 s/Cycle
E-51	EP-30	EP-45	EP-60	EP-75
TiO_2_ + E-51	TEP-30	TEP-45	TEP-60	TEP-75
NDZ-201/TiO_2_ + E-51	NTEP-30	NTEP-45	NTEP-60	NTEP-75
KH-560/TiO_2_ + E-51	KTEP-30	KTEP-45	KTEP-60	KTEP-75

**Table 3:** Kinetic parameters of isothermal curing for each curing system under Kamal model.

Curing System	*T*/°C	*k_1_*/min^−1^	*k_2_*/min^−1^	*m*	*n*
EP-60	75	0.00096	0.24	0.23	1.21
EP-60	85	0.0011	0.41	0.26	1.34
EP-60	95	0.0023	0.77	0.31	1.63
TEP-60	75	0.00091	0.25	0.25	1.31
TEP-60	85	0.0012	0.50	0.32	1.51
TEP-60	95	0.0017	0.96	0.39	1.69
NTEP-60	75	0.00095	0.18	0.19	1.37
NTEP-60	85	0.0011	0.43	0.29	1.48
NTEP-60	95	0.0015	0.92	0.35	1.78
KTEP-60	75	0.00076	0.23	0.25	1.22
KTEP-60	85	0.0011	0.43	0.28	1.34
KTEP-60	95	0.0017	0.89	0.37	1.67

**Table 4:** Isothermal curing activation energy of EP-60, TEP-60, NTEP-60, and KTEP-60 curing systems.

Curing System	E_a1_/kJ·mol^−1^	E_a2_/kJ·mol^−1^
EP-60	45.67	62.46
TEP-60	32.39	71.87
NTEP-60	25.52	88.35
KTEP-60	42.58	72.66

**Table 5:** Thermal decomposition temperature of each sample.

Composite	EP-60	TEP-60	NTEP-60	KTEP-60
*T*_5%/_°C	274.68	298.07	278.68	296.71
*T*_30%/_°C	364.50	367.99	368.42	368.35
*T_m/_*°C	364.17	363.65	378.80	373.92

## Data Availability

The data used to support the findings of this study are available from the corresponding author upon request.
